# A Biology-Driven Framework for Selecting, Engineering, and Validating Physiologically Relevant Multicellular Tumour Models

**DOI:** 10.3390/pharmaceutics18101227

**Published:** 2026-09-27

**Authors:** Ying-Ta Wu, Darshan T. Govindaraju, Girija M. Lakshmikanthacharya, Tzu-Yu Hsu, Chen-Fang Lin, Shashwat Sharma, Anilkumar T. Shivanna

**Affiliations:** 1 MatchCure Inc., 3F., No. 632, Guangfu South Road, Da’an District, Taipei City 10695, Taiwan; ywu@imatchcure.com (Y.-T.W.);; 2 Independent Researcher, Zhonghe District, New Taipei City 23561, Taiwan; 3 International Ph.D. Program in Medicine, College of Medicine, Taipei Medical University, Taipei City 11031, Taiwan

**Keywords:** physiologically relevant tumour models, multicellular tumour models, patient-derived models, tumour microenvironment, tumour organoids, tumour-on-chip, drug discovery, drug delivery, bioprinting, functional precision oncology

## Abstract

Physiologically relevant tumour models are increasingly used in cancer research, drug development, and functional precision oncology, yet selection often remains platform-led rather than biology-led. This structured critical narrative review proposes an operational framework linking model selection, engineering, and validation to six interdependent domains: multicellular organization and heterogeneity, extracellular-matrix architecture and mechanics, stromal regulation, vascular and interstitial transport, immune regulation and communication, and metabolic regulation with biochemical gradients. The framework distinguishes mechanism-guided questions, in which a candidate causal mechanism can be prespecified, from discovery-oriented questions, in which the determinant is unknown and candidate functions are tested through nested or factorial configurations before a minimal sufficient explanatory model is defined. Required functions are classified as essential, context-modifying, non-essential, or uncertain, and preservation-oriented, reconstruction-oriented, and hybrid strategies are distinguished from the technological format used to implement them. For pharmaceutical applications, delivered exposure, matrix or device binding, tissue penetration, transport, dosing schedule, and recovery are treated as qualification variables when they can change apparent potency or response. The practical contribution is a traceable biology-to-decision record linking the decision and mechanism status to function selection, model configuration, qualification evidence, complexity-retention rules, and claim boundaries. Model qualification is separated from external association, prospective predictive validity, and clinical utility. Among the representative studies appraised, direct functional and configuration-specific pharmacological evidence was demonstrated more often than interlaboratory transfer, independently validated prospective prediction, or clinical utility. Physiological relevance should therefore be demonstrated through decision-critical function rather than inferred from platform complexity or component count. The proposed decision rules remain unvalidated prospectively and across laboratories.

## 1. Introduction

Cancer research increasingly resolves tumours at genomic, single-cell, spatial, and functional levels, yet translation of promising preclinical findings remains inefficient [1,2]. Tissue engineering, patient-derived culture, and microphysiological technologies have expanded the available experimental systems [3], including spheroids [4], organoids [5,6], tumour-on-chip systems [7], and biofabricated tissues [8]. Most classifications begin with platform or fabrication technology, which can obscure the more consequential question: Which biological functions must be represented for a defined scientific or translational purpose?

This review evaluates tumour models by the functions they preserve or reconstruct rather than by technical sophistication. It links three tasks: define the functions required for a decision; preserve or engineer them in a controllable model; and qualify the resulting system for the intended claim. Engineering, automation, regulation, and future-development topics are included only where they affect reproducibility, measurement, or context of use. For pharmaceutical research and development, the same logic also requires separating pharmacodynamic biology from exposure determinants such as matrix or protein binding, tissue penetration, barrier transport, metabolism, device loss, and dosing schedule [9,10].

### 1.1. The Need for Physiologically Relevant Tumour Models

Despite advances in molecular profiling, targeted therapy, and immunotherapy, oncology drug development retains high clinical attrition [11]. Failure may reflect efficacy, toxicity, pharmacology, or resistance, and limitations of available preclinical models remain important in several disease and immuno-oncology settings [12]. A genotype-faithful system may still omit architecture, stroma, vascular delivery, immunity, or metabolic stress; its conclusions should not extend beyond the functions represented.

Solid tumours are spatially organized multicellular ecosystems in which malignant cells interact with fibroblasts, endothelial and perivascular cells, immune populations, extracellular matrix, and local biochemical and mechanical conditions [1,13,14]. Single-cell and spatial studies show that location and intercellular context shape cell state, invasion, immune evasion, metabolism, and treatment response [15,16]. Experimental relevance therefore requires preservation or reconstruction of the interactions governing the phenomenon under study.

Two-dimensional cultures remain indispensable for accessible, reproducible, and scalable experimentation [17] but simplify architecture, cell–cell and cell–matrix communication, gradients, and transport-dependent exposure [17,18,19]. Animal models retain systemic physiology and pharmacokinetics but are constrained by species differences, cost, throughput, and ethics [20]. New Approach Methodologies and microphysiological systems are therefore complementary tools rather than universal replacements [21,22,23]; for pharmaceutical questions, model choice should also distinguish cellular insensitivity from inadequate delivery or exposure.

No single alternative can replace every imperfect model. A monolayer may be optimal for a cell-autonomous mechanism, a spheroid for diffusion-dependent response, an explant for native architecture and resident immune cells, and a perfused system for endothelial transport or immune trafficking. Model selection should therefore begin with the decision to be supported and the minimum biological functions required to address it.

### 1.2. Evolution of Tumour Models Toward Physiological Relevance

Tumour modelling has expanded with the biological conception of cancer. Reductionist cultures established core principles of oncogenic signalling, proliferation, and drug sensitivity and remain essential. As tissue architecture and microenvironmental regulation became evident, model development broadened from maintaining malignant cells to reproducing selected functions of the environment in which they evolve [1,13,14].

Spheroids showed that aggregation can alter proliferation, diffusion, hypoxia, and treatment response [4]. Patient-derived organoids added renewable tumour-derived material for disease modelling and patient-specific experimentation [5,6]. Biomaterials and biofabrication enabled control of matrix and spatial organization, while tumour-on-chip systems introduced perfusion, barriers, and dynamic transport [3,7,8]. These developments expanded the functions that can be isolated, preserved, reconstructed, and measured; they did not create a simple hierarchy of model quality.

### 1.3. Defining Physiological Relevance in Multicellular Tumour Models

Physiological relevance is context-dependent rather than intrinsic to a platform. A model is relevant when it reproduces the functions required for a defined question with sufficient control for interpretation. The appropriate configuration depends not only on the intended decision but also on the epistemic state of the question. When a plausible decisive mechanism is already established, model selection can be mechanism-guided. When the purpose is to discover an unknown determinant of response or resistance, the mechanism must not be assumed in advance; candidate functions instead become experimentally testable variables. Physiological relevance, analytical performance, predictive validity, and clinical utility therefore require separate evidence. A mechanistically faithful model may be too variable for screening, while a simpler assay may predict a narrowly defined outcome.

Relevance is also distinct from maximal complexity. Added cells, matrices, sensors, or fluidic modules are justified only when they restore a required function, resolve a plausible alternative explanation, change an interpretation, improve validated performance, or enable a necessary measurement [2,21,22]. We refer to this as the minimal-sufficient-model principle. In mechanism-guided studies, it is an a priori design rule: begin with the least complex configuration capable of reproducing the prespecified causal function. In discovery-oriented studies, it is an a posteriori convergence rule: begin with a bounded set of plausible functions, test them through matched nested or factorial configurations, and define the minimal sufficient explanatory model only after the response-determining function has been localized and perturbed.

Conceptual contribution relative to existing approaches. Fit-for-purpose selection, modular model development, platform comparison, analytical qualification, and clinical validation are established ideas and are not claimed here as individually novel [2,21,22,24,25]. The specific contribution of this review is to connect them into an auditable two-entry decision framework. Each application generates a biology-to-decision record containing: (i) the decision and endpoint; (ii) whether the mechanism is established, suspected, or unknown; (iii) the classification of candidate functions as essential, context-modifying, non-essential, or uncertain; (iv) the model format, biological configuration, and preservation/reconstruction strategy chosen to represent those functions; (v) the qualification evidence and matched complexity test; and (vi) the supported and unsupported claim boundaries. The explicit complexity-retention rule makes this sequence practically testable while distinguishing absence of evidence from evidence of absence: an added module is retained when a prespecified comparison reproducibly changes mechanism, response ranking, uncertainty, or decision-relevant interpretation; simplification is supported only when the qualified comparison has sufficient precision and sensitivity to exclude a prespecified decision-relevant effect; otherwise, the result remains inconclusive and requires further testing.

Terminology used in this review. The six biological domains are multicellular organization and heterogeneity; extracellular-matrix architecture and mechanics; stromal regulation; vascular and interstitial transport; immune regulation and communication; and metabolic regulation with biochemical gradients. Three orthogonal descriptors are used for model selection. Model format denotes the physical or technological implementation (for example, spheroid, organoid, explant/slice, matrix-engineered or bioprinted construct, or tumour-on-chip/microphysiological system). Biological configuration denotes the compartments or functions represented within that format (for example, tumour-only, stromal, vascular, immune, metabolic/gradient-controlled, or integrated multicellular). Implementation strategy denotes whether the relevant biology is predominantly preserved, reconstructed, or combined in a hybrid. These descriptors are not mutually exclusive: an integrated multicellular configuration may be implemented in several technological formats.

### 1.4. Scope and Literature Approach

This structured critical narrative review used iterative PubMed/MEDLINE searches from database inception to 31 July 2026, supplemented by publisher records and citation tracking. Literature identification was purposive rather than protocol-registered or exhaustive; complete screening counts were not retained and cannot be reconstructed, and no quantitative pooling, formal risk-of-bias grading, or PRISMA flow diagram was undertaken. Searches combined the six biological domains with platform, application, validation, functional precision oncology, and comparative oncology terms. Representative concept blocks and appraisal fields are provided in Supplementary Methods S1 and Tables S1–S3. Because the approach was not systematic, retrieval frequencies were not used to estimate prevalence or the maturity of the field, and comparative conclusions are restricted to the representative evidence appraised. In response to peer review, targeted revision-stage PubMed verification searches for decellularized or patient-derived extracellular matrix, light-mediated bioprinting, and pharmaceutical exposure/drug-delivery modelling were rerun on 4 September 2026; scientific records were eligible only if published or electronically published by 31 July 2026. Exact targeted queries and the retained sources used for this revision are reported in Supplementary Table S10. The original narrative search was not prospectively logged query by query; therefore, complete historical query strings and screening counts are unavailable and were not retrospectively reconstructed. To improve reproducibility within that limitation, Supplementary Tables S1–S3 report the information sources and date coverage, representative Boolean concept blocks, and evidence-selection rules, whereas Tables S7 and S10 provide the exact date-stamped queries used for the targeted revision-stage commercial and scientific verification searches.

Original studies were prioritized for mechanisms, platform performance, treatment response, and translational association; reviews supported field-level concepts, while consensus, standards, and regulatory documents informed quality and adoption. Greater weight was given to direct functional measurement, matched configurations, mechanism-specific perturbation, patient-derived material, operational data, and independent benchmarks. Conflicting findings were interpreted by tumour type, specimen source, model configuration, exposure, endpoint, and validation design rather than pooled. Retained references underwent two distinct verification steps: bibliographic metadata were checked against PubMed and/or publisher records, and citation relevance was checked against the abstract or full text for the claim supported. A cross-document mechanical audit additionally checked citation-number continuity, undefined citations, uncited bibliography entries, and duplicate DOI records. Bibliographic correctness and claim-level relevance were treated as separate checks. These procedures increase transparency but do not constitute systematic-review screening or formal evidence grading; author-proposed operational rules are presented as testable recommendations and conclusions remain bounded to the representative evidence appraised.

Commercial platforms were assessed separately from the scientific-literature cutoff. The provider-specific evidence map was re-verified on 4 September 2026 using standardized exact provider/product-name searches combined with oncology terms, followed by publisher/DOI checks and review of official provider publication or application pages where available. A targeted PhysioMimix^®^ Core evidence recheck was performed on 15 September 2026 and incorporated into the commercial evidence map. Exact verification queries and evidence dispositions are reported in Supplementary Table S7. Provider webpages were also rechecked on 4 September 2026; provider sources were used only for architecture, availability, compatibility, format, and stated use, while journal articles and conference-level implementations were recorded separately with evidence provenance. The mapping remains descriptive, representative, non-exhaustive, brand-neutral, and non-ranking; providers were not contacted, unpublished information and MatchCure products were excluded, and full eligibility criteria and source boundaries are provided in Supplementary Tables S3, S5 and S7. These safeguards improve transparency but cannot eliminate affiliation-related selection or interpretation bias; the commercial map should therefore be read as a source-separated descriptive snapshot rather than an independent comparative evaluation. All exact provider/product queries reported in Supplementary Table S7 were re-run in PubMed/MEDLINE on 18 September 2026 for peer-reviewed journal literature. Publisher/DOI records and official provider publication/application pages were then used for source verification and, where applicable, for provider-reported or non-journal implementation evidence. A final commercial-platform and reference audit was completed on 22 September 2026. Commercial implementation references were rechecked against PubMed and/or publisher metadata, and citation-number continuity, duplicate DOI records, and bibliography-to-text citation coverage were re-audited before resubmission.

Evidence interpretation is anchored to the defined context of use and intended claim. Two linked evidence tracks are then used as non-cumulative evidence tags rather than as sequential stages. Track A records what has been demonstrated about the model itself—identity/structural fidelity, functional fidelity, and comparative pharmacological response evidence—whereas Track B records external or decision-performance evidence—external association, prospective predictive validity, and clinical utility. A study may satisfy any subset of domains supported by its data; no domain is automatically implied by another, and analytical/operational qualification is cross-cutting rather than a stage. Identity/structural fidelity requires direct evidence of identity, composition, or architecture/spatial correspondence; functional fidelity requires direct measurement of the required biological function and mechanism-specific perturbation for causal claims; and comparative pharmacological response evidence requires defined exposure, a justified matched comparator or configuration, and a reproducible response difference. External association requires comparison with an independent claim-matched benchmark. Prospective or retrospective outcome-association analyses using internally derived or retuned thresholds remain external-association/predictive-performance evidence and are not classified as prospective predictive validity. Prospective predictive validity is reserved for a locked assay configuration, analysis pipeline, endpoint, and prediction rule evaluated without retuning in an independent validation cohort. Clinical utility additionally requires a prespecified assay-guided decision strategy, evidence that the result changes management, and a clinically relevant decision or outcome benefit. Operational assignment rules are provided in Supplementary Table S8, with representative study-level applications in Supplementary Tables S4B and S6. Evidence labels in Supplementary Tables S4B and S6 were assigned and systematically verified by the corresponding author against the operational definitions in Table S8. Borderline or ambiguous cases were handled conservatively: a domain label was retained only when its minimum criterion was clearly supported; otherwise, the more conservative classification was used and the uncertainty was stated in the interpretive boundary. The final classification set was rechecked in full for internal consistency; no separate second-author or formal inter-rater verification was used.

Generative-AI support. OpenAI ChatGPT (GPT 5.6 Sol) was used as an assistive tool for manuscript organization, language and structural editing, and development of preliminary conceptual figure elements. Generative AI was not treated as a citable scientific source and was not used as an autonomous decision-maker for literature inclusion or exclusion, evidence classification, or scientific interpretation. Any AI-assisted output retained in the manuscript was checked against the underlying source material and reviewed by the authors, who remain responsible for the accuracy, interpretation, and final submitted content.

## 2. Biological Requirements of Physiologically Relevant Tumour Models

### 2.1. Biological Principles Underpinning Physiologically Relevant Tumour Models

The malignant phenotype reflects both tumour-intrinsic programmes and biological context. Three-dimensional architecture and cell–matrix interactions can alter malignant behaviour despite persistent oncogenic abnormalities [26,27]. Stromal, vascular, immune, matrix, and metabolic systems likewise regulate tumour state in context-dependent ways [1,13,14]. Context is functional, spatial, and temporal. Mechanical and stromal interactions can modify transport, nutrient availability, and treatment response [13,28,29]. Spatial profiling shows that invasive fronts and tumour–immune neighbourhoods contain distinct cellular states and interactions [15,16,30,31]. Single-cell studies further show that tumour and stromal programmes vary across patients and anatomical regions [32,33,34,35].

For this review, these principles are organized into six coupled biological domains (Figure 1): multicellular organization and heterogeneity; extracellular-matrix architecture and mechanics; stromal regulation; vascular and interstitial transport; immune regulation and communication; and metabolic regulation with biochemical gradients. The domains are functional descriptors rather than additive scores, and the decision-relevant unit may be a cell state, interaction, transport process, gradient, or treatment-dependent transition. Models of resistance or adaptation must also resolve change over time when temporal evolution is decision-critical. The six domains form an extensible backbone rather than a closed ontology; neural, endocrine, microbiome, metastatic-niche, or whole-body processes should be treated as additional modules when they are decision-critical, experimentally distinct, and associated with their own acceptance criteria.

Biology is also hierarchical: genotype constrains possible states, but tissue architecture and integrin-dependent organization can shift phenotype without changing the underlying oncogenic lesions [26,27]. Spatial neighbourhoods organize tumour–immune interactions [30,31]. Metabolic exchange and solid stress can couple compartments by changing nutrient availability and vascular function [28,29]. For model selection, the biologically relevant unit is therefore often an interaction or state transition rather than a cell type in isolation.

For patient-derived models, pre-analytical specimen state is a cross-cutting qualification input rather than a seventh biological domain (Figure 1). Dissociation, sectioning, and ex vivo processing can alter tissue integrity, cellular recovery, and stress responses [36,37,38,39], while culture conditions and continued propagation can shift clonal composition and measured drug-response heterogeneity [40,41,42]. Site or region, processing conditions, passage or cryopreservation history, and time to assay should therefore be prospectively documented as part of model qualification [24,25,43]. Where retention of fragile populations or spatial states is required, comparison with parental or time-zero tissue should be incorporated when feasible. A lost feature that is deliberately reintroduced is reconstructed rather than preserved; this distinction is used throughout the engineering, platform-selection, and validation sections.

### 2.2. Multicellular Organization and Heterogeneity

Solid tumours contain malignant and non-malignant populations whose biological influence depends on state and location as well as abundance. Spatial studies identify organized tumour–immune neighbourhoods at invasive fronts and within immune-rich or compartmentalized regions [30,31]. Single-cell studies reveal diverse malignant, fibroblast, and immune states within and across tumours [32,33,34,35].

Models should distinguish cellular composition from multicellular organization. Dissociation can perturb recovery and cell state [39], and reaggregation can recreate three-dimensional structure without preserving the original spatial architecture. Explants and precision-cut slices retain more native context initially, but this can drift during culture [36,37]. Patient-derived origin therefore does not itself demonstrate preservation of parental heterogeneity.

Tissue atlases define in vivo states and neighbourhoods [30,31], but model qualification requires direct comparison of decision-relevant states, relative abundance, or spatial relationships with parental or time-zero tissue when those features matter. Otherwise, the required interaction should be reconstructed and tested functionally rather than inferred from multicellularity alone.

Critical synthesis. Multicellularity is insufficient when cell state, spatial relationship, or interaction history is lost. Fidelity should therefore be defined at the level required by the question—state, abundance, spatial organization, or interaction-dependent phenotype—rather than by marker positivity alone.

### 2.3. Extracellular-Matrix Architecture and Mechanics

The extracellular matrix organizes tissue, transmits forces, presents ligands, and regulates transport. Integrin-dependent three-dimensional interactions can suppress malignant behaviour despite a persistent genotype [26], while polarized architecture and β4-integrin signalling can alter apoptotic response [27]. These experiments demonstrate that matrix context can change tumour-cell behaviour rather than merely provide physical support. Tumour matrix varies in composition, density, fibre alignment, cross-linking, degradability, and viscoelasticity. Increased matrix tension and cross-linking can enhance integrin signalling and malignant progression [44,45], while YAP/TAZ provides a mechanotransduction route through which extracellular forces regulate cell state [46].

Tumour and stromal cells reciprocally remodel the extracellular matrix. Cross-linking, solid stress, ligand presentation, and mechanotransduction can alter growth, survival, motility, vessel compression, transport, and treatment response [29,44,45,46]. Because these variables are coupled, a named hydrogel, bulk modulus, or collagen concentration is an incomplete surrogate; the decision-relevant matrix function should be isolated or measured directly where feasible.

Decellularized extracellular matrix (dECM) retains matrix-derived information while removing viable cellular compartments. Tumour- or patient-derived scaffolds and hydrogels can preserve tissue-specific composition, architecture, mechanics, and bioactive cues, and matrix source can alter transcriptional state and chemotherapy response [47,48,49]. These systems are best interpreted as preserved matrix information combined with reconstructed cellular context, not as preserved intact tumour microenvironments.

Decellularization is itself a pre-analytical intervention: detergents, enzymes, mechanical processing, solubilization, sterilization, and cross-linking can alter matrix composition, porosity, ultrastructure, mechanics, or soluble cues, while residual cellular material or reagents may affect recellularization [50]. Patient-derived origin therefore does not establish matrix fidelity; retained matrisome and decision-relevant mechanical or transport properties should be benchmarked to source tissue where feasible.

Critical synthesis. Matrix fidelity cannot be reduced to material name or one mechanical value. Composition, mechanics, remodelling, and transport should be qualified at the level required by the claim, with remaining confounders stated explicitly.

### 2.4. Stromal Regulation

Tumour stroma comprises multiple non-malignant cell states and extracellular components that regulate tumour behaviour through reciprocal signalling. Fibroblast heterogeneity is particularly well established, with inflammatory, myofibroblastic, and antigen-presenting states described in pancreatic cancer [35,51]. Cancer-associated fibroblasts can regulate matrix, cytokine and growth-factor signalling, metabolism, and therapeutic response [28,51,52]. Their effects are conditional because fibroblast states differ by tumour, patient, and region [35,51], rather than constituting a uniformly tumour-promoting population.

Pancreatic-cancer studies illustrate stromal conditionality: broad depletion of alpha-smooth-muscle-actin-positive fibroblasts or Sonic-hedgehog-dependent stroma produced more aggressive disease, greater immunosuppression, or reduced survival [53,54]. Stromal states can also support metabolism [28], alter chemotherapy response [52], modify mechanics [55], and impair transport through solid stress [29]. Thus, stromal effects cannot be assigned from fibroblast abundance alone.

Generic fibroblast inclusion is not evidence of stromal relevance. Models should define stromal origin and state, justify component ratios, and demonstrate a conditional effect on a prespecified tumour, matrix, immune, metabolic, or pharmacological endpoint (Table 1) [28,35,51,52,53,54].

Critical synthesis. Stromal relevance requires defined origin and state plus a demonstrable effect on the endpoint being studied; a broad stromal marker cannot be assumed to indicate a uniform direction of effect [53,54].

### 2.5. Vascular and Interstitial Transport

Tumour vasculature is both a transport system and a regulatory tissue. Angiogenesis supports tumour growth [71], but tumour vessels can contain defective endothelial junctions and exhibit abnormal permeability and perfusion [56,58]. These abnormalities can alter tissue delivery of oxygen and therapeutics [57,58,72]. Vascular function, not vessel density, is the decisive variable for transport. VEGFR2 blockade can transiently normalize vascular function, reduce pressure barriers, and improve tumour penetration or oxygenation [57,72], which underpins the vascular-normalization concept [58].

Endothelial cells are not a uniform compartment. Single-cell and organotypic studies identify tumour- and tissue-specific endothelial programmes [73,74,75,76], while vessel co-option provides a non-angiogenic vascular strategy that can contribute to resistance to antiangiogenic therapy [77,78]. Vascular physiology is also coupled to mechanics because growth-induced solid stress can compress vessels [29].

Evidence from tissue and in vivo studies does not automatically qualify an engineered vascular compartment. The claimed function—perfusion, permeability, barrier behaviour, tissue delivery, or trafficking—must be measured directly [59,60]. Endothelial-marker expression or vessel-like morphology is insufficient, and tumour- or tissue-specific endothelial state should be considered where it is decision-critical [73,74,75,76].

Critical synthesis. Vascular relevance should be assigned to the measured transport function, not endothelial inclusion or vessel appearance. Delivered-dose measurements are essential when perfusion, permeability, pressure, or device loss can mimic biological resistance [9,59,60].

### 2.6. Immune Regulation and Communication

Immunosurveillance and cancer immunoediting established that immunity can eliminate transformed cells, restrain tumour growth, and select escape variants [61,62,79]. Tumour progression therefore occurs under continuing immune selection rather than in isolation from host immunity. Within established tumours, lymphoid, myeloid, stromal, endothelial, and malignant compartments communicate through cytokines, chemokines, metabolites, antigen presentation, and cell contact [13,63]. Computational ligand–receptor methods can nominate candidate intercellular interactions [80,81,82], but those predictions require functional validation before being treated as causal communication.

Immune populations occupy diverse and plastic states [34,83,84], and tumour, stromal, vascular, matrix, and metabolic context can alter both activation and tissue access [13,29,30,31,63]. Direct juxtaposition of activated lymphocytes and tumour cells therefore measures effector capacity after contact, not recruitment or entry. Organoid and tumour-on-chip systems can resolve distinct stages—recognition, migration, endothelial crossing, infiltration, engagement, and killing—when stage-specific readouts and controls are used [64,65,85,86,87,88].

Critical synthesis. Direct tumour killing can validate effector function while remaining uninformative about recruitment, tissue entry, suppression, or persistence. Claims should therefore be restricted to the immune step reconstructed and measured.

### 2.7. Metabolic Regulation and Biochemical Gradients

Tumour metabolism is heterogeneous, spatially organized, and microenvironment-dependent. Warburg’s aerobic-glycolysis framework remains foundational [89], but human isotope-tracing studies demonstrate interpatient and intratumour variation in glucose oxidation and lactate use [66,67], while spatial multi-omics identifies region-specific metabolic programmes and tumour–non-tumour interactions [90]. Metabolic behaviour therefore cannot be inferred from a single pathway or genotype. Abnormal perfusion, diffusion distance, cell density, and matrix structure generate overlapping gradients of oxygen, nutrients, pH, and metabolites [68,91,92]. These gradients are not interchangeable because each reflects a different balance of delivery, consumption, diffusion, and clearance.

Cells adapt through metabolic plasticity and intercellular exchange: pancreatic stellate cells can supply alanine to tumour cells [28], while tumour cells can compete with T cells for nutrients [69]. Metabolic models should therefore reproduce and measure the relevant substrate, gradient, flux, or exchange process rather than infer it from generic three-dimensional culture. Spatial or sensor-based methods can map oxygen, pH, and metabolites [68,90,93,94,95], whereas flux claims require direct transport or isotope-tracing measurements [66,67].

Critical synthesis. Metabolic relevance requires separating state from flux and cause from consequence. Construct size, a hypoxia marker, or a bulk metabolite measurement is only a surrogate unless the decision-relevant gradient or exchange process is quantified and, for causal claims, perturbed [68,69,91].

Integrated interpretation. The six domains in Figure 1 are coupled causal functions rather than an additive checklist: matrix and solid stress can alter vessels [29], vascular function shapes biochemical gradients [68,72], stromal programmes influence matrix and immunity [51,63], and metabolic competition can suppress antitumour immunity [69]. Their relevance should therefore be judged by whether the required function is represented and qualified through decision-relevant outputs such as growth, invasion, drug response, resistance, or another claim-matched endpoint. More represented domains do not automatically increase physiological relevance; the preferred configuration is the shortest defensible causal chain linking the decision-critical biology to the endpoint (Table 1; Figure 1).

## 3. Engineering Strategies for Reconstructing Physiologically Relevant Tumour Models

### 3.1. Translating Biological Requirements into Engineering Design

Engineering translates a biological question into controllable variables, acceptance criteria, and a bounded claim. The intended decision should determine cell sources, organization, matrix, transport, timing, exposure, and readouts. A feature is retained only when it changes the relevant state, resolves an alternative explanation, or improves fitness for the context of use. At the glioblastoma margin, Cornelison et al. combined patient-derived glioma stem cells, astrocytes, microglia, and interstitial flow. Flow and glial composition altered invasion, proliferation, and stem-like behaviour [96]. The matched perturbations support functional fidelity; comparison with xenograft survival provides an external benchmark, not patient-level prediction.

A pancreatic-cancer chip combined patient-derived organoids, stellate cells, macrophages, and perfusion. The multicellular configuration increased collagen deposition and invasion, and stroma-modulating agents enhanced chemotherapy only in that context [97]. Tumour-only comparisons therefore support functional and comparative pharmacological response evidence, not clinical prediction. Design strategies remain question-specific. A spheroid-on-chip isolated flow–hypoxia interactions [98], whereas a perfused tumour-slice chip preserved native organization for transport and longitudinal analysis [99]. Automated bioprinting can improve repeatable manufacture [100], but scale does not establish biological or predictive validity. 

Operational model-selection sequence. The framework has two entry routes. In a mechanism-guided study, define the decision, endpoint, claim boundary, and timeline; qualify specimen provenance and culture history; specify the candidate decisive mechanism; classify the functions required to represent that mechanism; map them to the six domains and any necessary external module; assign readouts and acceptance criteria; and begin with the minimal sufficient configuration. Additional modules are retained only when matched comparisons provide decision-relevant information. Discovery-guided route: When the determinant of response, resistance, or adaptation is unknown, the framework does not require a decisive mechanism to be named in advance. The starting point is the phenotype or decision uncertainty, followed by a bounded set of plausible functions derived from the disease context and available evidence. Those functions are treated as experimental variables in nested or factorial configurations—for example, tumour-only versus tumour–stroma, altered matrix, perfused, or combined conditions under otherwise matched exposure. A response shift localizes the relevant compartment or process; targeted perturbation is then required before a mechanistic claim is made. The minimal sufficient explanatory model is defined after this discovery step and becomes the configuration used for confirmation, transfer, or prediction.

Convergence and stop rule. Both routes then proceed through the same four operations: specify the model using the orthogonal descriptors of model format, biological configuration, and implementation strategy; define claim-specific identity/structural, functional, and analytical/operational qualification criteria; test whether added complexity reproducibly changes the mechanism, response ranking, uncertainty, or decision-relevant interpretation; and match the demonstrated evidence to the intended claim. The complexity test has three possible outcomes. An added module is retained and requalified when a reproducible decision-relevant effect is demonstrated. It may be removed and the model simplified only when the comparison is adequately qualified and sufficiently precise and sensitive to exclude a prespecified decision-relevant effect. When assay performance, uncertainty, sample size, exposure control, or measurement sensitivity does not permit either conclusion, the result is inconclusive and the necessity of the module remains unresolved pending additional testing. The final interpretation is a supported, bounded claim, with unsupported extrapolations stated explicitly. This sequence is an author-proposed decision rule rather than a validated standard.

Classification of candidate functions and engineering translation. Essential functions are required for a prespecified inference; context-modifying functions may alter magnitude or ranking without being necessary for the core inference; non-essential features add burden without changing the decision under the tested conditions; and uncertain functions remain explicit candidates for sensitivity analysis or nested-model testing. Figure 2 translates the six biological domains into an engineering matrix rather than a ranked platform list or sequential workflow. For each domain, it links the decision-critical biological requirement to configurable model variables, illustrative implementation options, and qualification evidence. Multicellular organization is therefore specified through cell identity, relative abundance, spatial arrangement, and state; matrix biology through composition, mechanics, architecture, and degradability; stromal biology through cell origin/state and reciprocal cues; vascular biology through flow, barrier properties, and delivered exposure; immune biology through source, compatibility, state, route, and timing; and metabolic biology through oxygen, nutrient, pH, metabolite, and regional supply–consumption conditions. Qualification should directly test the corresponding structure or function rather than infer success from component presence alone. Repeatability, controls, pre-analytical robustness, delivered exposure/recovery where relevant, endpoint stability, assay-failure denominators, and workflow/version control are cross-cutting analytical/operational requirements. The examples in Figure 2 are illustrative rather than prescriptive; not every variable, implementation option, or measurement is required in every model.

**Worked applications.** Box 1 applies the mechanism-guided route to adoptive-cell-therapy evaluation. Box 2 provides two contrasting hypothetical pharmacology examples: one in which the framework supports de-escalation to a simpler model, and one in which controlled increases in complexity are used to discover an unknown resistance determinant. These examples illustrate decision logic; they are not evidence that the framework itself has been validated.

Box 1 **Worked application of the biology-driven framework: adoptive-cell-therapy evaluation. The table is intentionally organized as a short decision workflow rather than a comprehensive protocol.**

**Step**

**Application to Adoptive-Cell-Therapy Evaluation**
1. DecisionDistinguish failure of recognition/killing from failure of tissue access or post-entry suppression.2. Start modelAntigen-compatible 3D tumour + defined engineered T-cell product; resolve contact, killing and persistence over time [86,87].3. QualifyTumour/antigen and T-cell identity; viability/activation; reproducible architecture; target engagement; apoptosis or durable control; regrowth.4. ControlsTarget-positive/negative; tumour-only; immune-only; vehicle/blockade; control separation, replicate precision and a prespecified assay window.5. Test uncertaintyAdd matrix, fibroblast, myeloid or endothelial modules only when exclusion, trafficking or suppression is a plausible explanation.6. Escalate/retainUse endothelial modules for adhesion/transmigration/delivery claims [65]; retain stromal/myeloid modules only when matched models change access, cytotoxicity or ranking [88,101,102,103].7. Claim boundarySupports stage-specific recognition/killing, access or suppression; not organism-level PK, systemic immune priming/toxicity, prospective patient response or clinical utility.

Box 2 **Contrasting applications of the two-entry framework: mechanism-guided de-escalation and discovery-guided escalation.**

**Decision Record**

**Mechanism-Guided Pharmacology**

**Discovery-Oriented Resistance**
DecisionDoes target-directed activity persist in a qualified 3D model at defined exposure?Why is activity lost after tissue-context reconstruction?Mechanism statusCandidate tumour-cell mechanism established or strongly supported.Determinant unknown; stroma, matrix, transport or other functions remain plausible.Starting designQualified spheroid/organoid with exposure and tumour-response controls.Tumour-only baseline + matched nested modules under the same exposure and endpoint.Complexity testAdd modules only to test a plausible alternative explanation.Add/remove candidate modules; localize the response shift, then perturb the implicated process.Stop ruleRetain when the added module reproducibly changes mechanism, ranking, uncertainty, or interpretation; simplify only when a qualified comparison can exclude a prespecified decision-relevant effect; otherwise, classify the result as inconclusive.Retain modules needed to reproduce and causally perturb the discovered effect. If precision, sensitivity, exposure control, or assay performance is insufficient to resolve a candidate module, classify it as inconclusive and retest rather than removing it.OutputA simpler model is supported only when the qualified comparison excludes a prespecified decision-relevant contribution from the added module; otherwise, its necessity remains unresolved.The minimal sufficient model is defined after discovery only after informative, non-informative, and inconclusive modules have been resolved sufficiently for the intended claim.

Biology-to-decision record. For each application, the framework records the decision and endpoint, mechanism status, candidate-function classification, model format/configuration/strategy, qualification evidence, matched complexity test, retention or stop rule, and supported/unsupported claim. A reusable blank template is provided in Supplementary Table S9.

### 3.2. Engineering Extracellular-Matrix Architecture and Mechanics

Extracellular-matrix engineering converts matrix biology into controllable variables: composition and ligand presentation, stiffness and stress relaxation, fibre architecture and porosity, and degradability. These variables affect receptor engagement, mechanotransduction, migration, diffusion, and remodelling, so matrix choice should be part of the experimental hypothesis rather than a neutral culture condition. Naturally derived matrices provide endogenous cues but can couple composition and mechanics and introduce lot variation.

dECM can be used as intact or sectioned scaffolds, particulate material, solubilized hydrogels, or printable bioinks. Intact scaffolds retain more native topology, whereas solubilized formats improve handling, concentration control, microfluidic compatibility, or biofabrication. Patient-derived colorectal, ovarian, and cholangiocarcinoma matrices show that source-specific cues can alter tumour state and chemotherapy response [47,48,49].

dECM qualification should report tissue source, decellularization and downstream processing, residual DNA/cellular material, retained structural or matrisome features, mechanics, porosity, and batch/patient variability [50]. For pharmacological use, matrix binding, diffusivity, recovery, and spatial exposure should be measured when they can alter apparent potency. Matched patient-derived ECM and organoids remain a hybrid preservation/reconstruction system requiring separate qualification of matrix, cells, and their interaction.

Engineered and synthetic matrices can improve control or separate variables. EKGel retained selected patient-derived breast-organoid features while providing tuneable mechanics and lower batch variability [104]; a cervical ECM hydrogel altered establishment and behaviour [105]. Synthetic hydrogels can independently tune ligand presentation, stiffness, and degradability, with reported effects on tumour growth, fibroblast interactions, dormancy-associated drug response, and printability [106,107,108]. These findings remain configuration-specific.

Reporting should match intended use: invasion models require remodelling measures; transport studies require porosity, binding, and diffusivity; and drug testing requires control of phenotype and compound availability. Matrix identity/source, composition, mechanics, degradation, remodelling, and relevant exposure variables should be reported.

Critical synthesis. Matrix composition and mechanics can alter tumour state and treatment response, but most evidence remains configuration- and laboratory-specific [104,105,106,107,108]. Stronger evidence requires matched material perturbations, direct measurement of the claimed function and compound availability, and transfer testing under intended operating conditions [24,43].

### 3.3. Engineering Multicellular Organization and Heterogeneity

Engineering multicellular architecture requires control of composition, abundance, spatial proximity, compartment boundaries, and interaction timing. The relevant unit is the interaction produced, not the number of cell types mixed. Self-assembly can generate informative but variable organization; in one carcinoma model, tumour-generated compression drove fibroblasts into an encasing layer [109].

Organoid–stromal assembloids restore interactions lost during conventional expansion. Fibroblasts reorganized around oesophageal tumour organoids and altered tumour states [110], while controlled lung-cancer microgels retained patient-derived populations, fibroblast-state variation, configuration-specific treatment differences, and concordance between ex vivo drug responses and clinical outcomes in a seven-patient comparison [111]. These findings support structural and functional evidence; for the lung-cancer assembloid, the small outcome comparison supports limited external association rather than independently validated prospective prediction. Bioprinting and microfabrication can make location an experimental variable by placing tumour, stromal, endothelial, and immune cells in defined compartments. A bioprinted breast-tumour-on-chip linked local responses to controlled chemical gradients [112]. Imposed geometry, however, may restrict the migration and remodelling that would otherwise emerge.

The assembly sequence should reflect the biological process rather than fabrication convenience. Simultaneous seeding can model coordinated formation, whereas sequential addition can model recruitment, infiltration, angiogenesis, or metastatic colonization and may prevent rapidly proliferating populations from dominating. Quality control should distinguish standardized inputs from biological outputs such as state, spatial rearrangement, and differential response.

Critical synthesis. Composition, geometry, and assembly sequence are experimental variables, and engineered architecture may reflect fabrication as much as parental tissue [109,110,111,112]. Added cell types should be retained only when matched configurations change a prespecified phenotype, mechanism, or response.

### 3.4. Engineering Vascular and Interstitial Transport

Vascular engineering should be defined by the required transport function—lumen continuity, flow, shear, barrier integrity, permeability, interstitial transport, trafficking, or intravasation. Predefined endothelialized channels provide controlled access to shear, pressure, permeability, and dosing; self-assembled networks capture capillary morphogenesis but are less geometrically predictable; hybrid systems combine engineered access with tissue-scale microvascular beds.

Nashimoto and colleagues connected a tumour spheroid to a perfusable microvascular network, increasing growth and producing heterogeneous tracer and drug delivery [59]. A second platform imposed well- and poorly perfused geometries that generated distinct concentration profiles, drug responses, and endothelial toxicity [60]. Together, these studies establish transport-dependent response within the tested configurations.

A vascularized colorectal microtumour reproduced selected in vivo expression and treatment responses better than two-dimensional or non-vascularized controls [113], while an omentum-on-chip model captured tumour-induced endothelial and mesothelial barrier disruption [114]. Validation should match the claimed vascular function and include lumen continuity, permeability, pressure/flow, tissue transport, and compound recovery as relevant. Device absorption can separate nominal from delivered concentration [9].

Required reconstruction varies by question: tissue maintenance may need exchange alone; drug delivery requires perfusable lumens and quantified barrier and tissue transport; angiogenesis requires sprouting; immuno-oncology requires adhesion and transmigration; and metastasis may require invasion through extravasation. Section 3.1 provides the decision rule.

Critical synthesis. Perfusion and barrier configuration can alter delivered exposure and apparent response, but evidence remains specific to geometry, material, flow regime, and compound [59,60,113,114]. Vascular pharmacology therefore requires direct transport and compound-recovery measurements, with interlaboratory benchmarking still a priority [9,24,43].

### 3.5. Engineering Immune Regulation and Communication

Immune engineering should reconstruct the stage that determines the endpoint, including cell source, antigen compatibility, activation, route of entry, migration, endothelial crossing, target engagement, persistence, or adaptation. Immune-cell addition alone is not a functional model. Selection of the immune-cell source should follow the mechanism. Peripheral-blood, tumour-infiltrating, engineered T-cell, myeloid, NK-cell, and antigen-presenting-cell sources represent different biological states and should be selected according to the immune mechanism being modelled [63]. Availability alone can create a biologically mismatched immune model.

Culture compatibility is a major constraint. In cholangiocarcinoma organoid–immune co-cultures, supplements that supported organoid growth inhibited T-cell proliferation and activation; medium modification restored immune function and enabled tumour killing [64]. In a pancreatic organoid-in-matrix platform, T-cell killing varied with the extracellular environment, showing that failed cytotoxicity may reflect restricted migration or contact rather than deficient recognition [85]. Medium and matrix are therefore active determinants of immune function, not neutral background conditions.

Dynamic readouts separate mechanisms that endpoint viability conflates. BEHAV3D resolved productive and ineffective engineered-T-cell behaviours in patient organoids [86], while a colorectal chip separated migration, contact, and killing and linked APC loss to impaired engagement [87]. These studies provide mechanistic discrimination, not external prediction. Vascularized systems extend immune modelling to tissue entry. A breast-cancer chip resolved CAR-T-cell adhesion, transmigration, infiltration, killing, and endothelial injury [65], while patient-derived lung chips showed sample-specific anti-PD-1-associated killing and fibroblast-mediated suppression [88]. These are functional and comparative response studies; title terms such as ‘patient-specific’ or ‘personalized’ do not establish prospective predictive validity or clinical utility.

Immune integration may reconstruct defined components or preserve endogenous tissue. Reconstitution improves control but isolation and expansion can alter phenotype; explants, fragments, and air–liquid-interface cultures retain native immune architecture only transiently. Choice should follow whether the study requires manipulation, patient-specific recognition, acute checkpoint response, or longer-term adaptation. Assay duration, cytokine support, and cell-introduction sequence should match the mechanism. Short assays capture migration and acute killing; longer studies address exhaustion, antigen loss, suppressive reprogramming, and adaptive resistance but increase culture-induced drift. Corresponding quality measures are listed in Table 1 and Section 6.2.

Critical synthesis. Current models are strongest for acute, stage-resolved questions such as recognition, migration, contact, and killing [64,65,85,86,87,88]. Evidence is less mature for sustained exhaustion, antigen evolution, myeloid reprogramming, systemic priming, and immune toxicity. Allogeneic recognition, cytokine-driven overactivation, culture-induced drift, and endpoint-only viability can generate misleading activity. Immune models therefore require compatibility controls, longitudinal functional readouts, and explicit restriction of claims to the reconstructed immune stage.

### 3.6. Engineering Integrated Multicellular Tumour Microenvironments

Matrix, stromal, vascular, immune, and metabolic processes are coupled; integrated models are justified when reciprocal interactions alter tissue function or the experimental conclusion. Integration should be judged by measurable coupling rather than component count, and assembly may need temporal staging to model recruitment, angiogenesis, infiltration, colonization, or treatment-induced reorganization [115].

Longitudinal sensing can expose adaptation missed by terminal assays, as illustrated by embedded pH sensing in a pancreatic tumour–stroma model [93]. More generally, a baseline tumour–matrix system can be extended with stromal, vascular, immune, or sensing modules, with each addition tested by matched comparison and followed by requalification of medium, matrix, timing, transport, exposure, and endpoints.

Critical synthesis. Integrated systems are retained when adequately qualified matched comparisons demonstrate decision-relevant information unavailable from a simpler configuration. Failure to detect such an effect is not sufficient to justify simplification when experimental precision or sensitivity cannot exclude a meaningful contribution; in that case, the result remains inconclusive and the module should be requalified or retested. Because added modules introduce new failure modes and analytical burden, integration should use nested configurations, module-specific controls, measured coupling, and explicit stop rules [24,43,93,116].

## 4. Representative Physiologically Relevant Multicellular Tumour-Model Platforms

Model selection should not collapse model format, biological configuration, and implementation strategy into a single platform taxonomy. These are orthogonal, context-dependent descriptors (Figure 3). Model format denotes the physical or technological implementation—spheroid, tumour organoid, explant/slice, matrix-engineered or bioprinted construct, or tumour-on-chip/MPS. Biological configuration denotes the compartments or functions represented within that format, ranging from a tumour-only baseline to stromal, vascular, immune, metabolic/gradient-controlled, or integrated multicellular configurations. Implementation strategy denotes whether the decision-critical biology is predominantly preserved, reconstructed, or combined in a hybrid. Thus, an integrated multicellular configuration is not a sixth platform class: it can be implemented across several model formats. Two-dimensional monolayers remain useful external comparators but are not part of this multicellular format set.

Preservation-oriented strategy. Explants, slices, fragments, and some minimally dissociated clusters most directly retain short-term native neighbourhoods, matrix, resident immunity, or regional pathology. Preservation is a strategy rather than a unique platform property: preserved tissue can also be loaded into a chip or other engineered support. Interpretation requires parental- or time-zero comparison and evidence that the required compartments remain stable during the assay. Sampling bias, injury, ex vivo drift, finite tissue, and loss of systemic input are characteristic limitations.

Reconstruction-oriented strategy. Spheroids, conventional organoids, engineered matrices, bioprinted constructs, and many microphysiological systems rebuild selected functions after dissociation or de novo assembly. Reconstruction provides perturbability, standardization, expansion, and longitudinal analysis, but establishment, seeded ratios, medium, matrix, geometry, or device conditions may generate states absent from the parental tissue. The same technological format can therefore support several distinct biological configurations.

Hybrid strategy and selection rule. Hybrid models combine the retained patient-derived state with reconstructed functions when a decision-critical process is missing, such as perfusion, immune trafficking, or controlled stromal interaction. Retained tissue, added modules, and their coupling require separate qualification. Table 2 summarizes the decision-oriented capabilities and limitations of the five major model formats, while Figure 3 separates those formats from cross-cutting biological configurations and from preservation, reconstruction, and hybrid implementation strategies.

### 4.1. Multicellular Tumour Spheroids

Multicellular tumour spheroids are scalable reconstruction-oriented models of three-dimensional organization. Aggregation restores cell–cell contacts and diffusion distances that can alter proliferation, oxygenation, metabolism, drug penetration, and response relative to monolayers [70,117,118]. The category spans distinct systems. Homotypic cell-line spheroids mainly model tumour architecture and diffusion; heterotypic spheroids reconstruct selected stromal, endothelial, immune, or neural interactions; and patient-derived aggregates may retain interpatient variation or selected native compartments. Their relevance depends on cell source, processing, retained composition, and demonstrated function rather than spherical morphology alone [70,118,119].

Size, compactness, composition, growth, and internal viability must be qualified because larger aggregates can develop proliferative, quiescent, hypoxic, or necrotic regions. In heterotypic spheroids, the final organization may differ from the seeded ratio [70,118]. Added cell types are informative only when matched configurations change a defined phenotype, mechanism, or pharmacological response, as shown for fibroblast-containing ovarian-cancer spheroids [118].

Evidence for spheroids is strongest for controlled three-dimensional growth, diffusion-dependent response, and modular multicellular comparison. Patient-derived spheroids permit rapid testing but are limited by dissociation selection, finite yield, and laboratory-specific workflows. Preliminary clinical association has been reported [119], but claims requiring native architecture, vascular delivery, systemic metabolism, or sustained immune recruitment need additional qualified modules [24,43,70,118,119,120,121].

### 4.2. Tumour Organoids and Patient-Derived Organoid Systems

Tumour organoids are expandable reconstruction-oriented cultures whose principal advantage is renewable patient-derived material for repeated experiments, cryopreservation, genetic manipulation, profiling, and biobanking [122,123,124,125]. The term should be qualified by configuration because expandable epithelial organoids, short-term intact organoids, air–liquid-interface cultures, stromal or immune assembloids, and organoid-on-chip systems preserve different biological functions.

Establishment is also a selection process. Sampling, dissociation, matrix, medium, formation efficiency, passage, and cryopreservation determine which populations expand. Pancreatic organoids retained disease-related niche-factor dependencies [122], whereas breast- and bladder-cancer biobanks preserved major tumour features, with culture-associated evolution reported particularly in the bladder-cancer series [123,124]. These findings support identity and functional characterization, not patient-response prediction.

Conventional expandable organoids mainly retain malignant epithelium; stroma, endothelium, immune cells, and native matrix are usually depleted. Co-cultures, assembloids, air–liquid-interface cultures, and organoid-on-chip systems can restore selected functions, but each configuration introduces different media, matrix, ratio, and stability requirements [110,111,126]. Evidence obtained for one organoid configuration should not be transferred to another without requalification.

Clinical-response studies provide treatment-specific external association or predictive-performance evidence of varying strength [127,128,129,130,131]. Interpretation depends on study design, assay-success denominator, treatment allocation, threshold independence, and exact configuration (Supplementary Tables S4A, S4B and S6). These studies do not establish general clinical utility, and intact versus reformed organoids may require different analytical controls.

Organoids are best supported for renewable disease modelling, biobanking, biomarker discovery, and tumour-cell-directed pharmacology. Limitations include selective establishment, non-epithelial loss, matrix-/medium-dependence, normal-cell overgrowth, delayed availability, and variable success [122,123,124,125,126,127,128,129,130]. Predictive performance is disease-, treatment-, threshold-, and workflow-specific rather than an inherent platform property.

### 4.3. Tumour Explants, Precision-Cut Slices, and Minimally Dissociated Patient-Derived Models

Explants, precision-cut slices, intact fragments, and minimally dissociated clusters are predominantly preservation-oriented. At collection they can retain native matrix, multicellular composition, vascular remnants, and spatial relationships, but preservation at time zero does not guarantee stability during culture [36,37].

Formats trade preservation for experimental control. Explants retain broad architecture but vary in size; slices improve thickness consistency and parallel treatment; and smaller fragments or clusters increase throughput while progressively disrupting native organization. Anatomical origin, processing interval, cutting or dissociation method, thickness, and culture support are therefore part of the model definition [36,37,38].

Explants provide short-term access to endogenous stromal, immune, and matrix relationships and support pharmacodynamic, spatial, and immune analyses [36,132,133,134,135]. However, excision removes perfusion and systemic input, cutting changes diffusion, and injury or inflammatory drift can arise within days [37]. Compounds are delivered by ex vivo media rather than intact pharmacokinetics, so the usable assay window and exposure route require direct qualification.

### 4.4. Engineered Matrix-Based and Bioprinted Tumour Models

Engineered matrix-based and bioprinted models are reconstruction-oriented systems that make matrix composition, stiffness, degradability, porosity, geometry, and cell position experimental variables [112,136]. They are most informative when physical or spatial context is part of the causal hypothesis rather than a background culture condition.

Matrix formulations can couple composition, mechanics, and printability, while biological extracts and dECM can retain tissue-specific signals but introduce source- and processing-dependent variability [47,48,49,50,104,105,108]. Synthetic matrices permit more independent control of stiffness, ligands, and degradation [106]. Matrix properties can induce dormancy or modify treatment response [107], so material selection must be justified by the biological state and measured function required. dECM scaffolds and hydrogels occupy an intermediate position between preservation and reconstruction: they may retain aspects of the source matrix while the viable cellular compartments and culture conditions are rebuilt.

Bioprinting is not synonymous with extrusion. Extrusion-based approaches provide flexible multi-material deposition and broad bioink compatibility but expose cells and materials to nozzle geometry, shear, pressure, and rheological constraints [112,136,137,138,139]. Light-mediated vat-polymerization approaches, including stereolithography (SLA) and digital light processing (DLP), instead spatially photopolymerize photocurable bioinks without nozzle extrusion; SLA typically scans a focused light source, whereas DLP projects a patterned image across an entire layer [140]. These approaches can provide high spatial resolution and rapid fabrication of channels or patterned microenvironments, but they introduce different constraints involving photoinitiator chemistry, wavelength and light dose, optical attenuation, oxygen inhibition, cell density, and post-cure network properties.

Cancer applications illustrate the distinct questions enabled by light-based fabrication. A DLP-printed perfusable non-small-cell lung-cancer construct was used to compare gemcitabine response under static and dynamic culture, demonstrating strong dependence of apparent drug response on hydrogel penetration and perfusion [141]. DLP has also been used to fabricate tumour-derived breast-cancer models with vessel-like channels for drug-screening comparisons [142]. These studies support fabrication and configuration-specific pharmacological evidence; they do not establish that DLP or SLA is intrinsically more physiological than extrusion or another format.

Patient-derived tissues add variability in viability, matrix production, growth, and fabrication tolerance. Miniaturized microgels or assembloids may improve input standardization [111], while patient-derived dECM can add tissue-specific information when retained composition and mechanics are qualified [47,48,49]. Evidence is strongest for mechanobiology, invasion, spatial co-culture, transport, and secondary mechanistic testing; patient-level prediction and interlaboratory transfer remain limited. Drug studies should characterize matrix binding, diffusion, recovery, delivered exposure, and relevant photochemistry or post-cure properties.

### 4.5. Tumour-on-Chip Systems and Cancer Microphysiological Systems

Tumour-on-chip systems are a subset of cancer microphysiological systems [7,143,144]. They combine tumour tissue with defined compartments, controlled transport, and analytical access. Depending on the loaded tissue and modules, they may be reconstruction-oriented or hybrid. The device is infrastructure rather than a biological model by itself [116,145].

Compartmentalization can separate soluble signalling from direct contact, vascular delivery from tumour-cell sensitivity, and sequential immune processes such as adhesion, extravasation, migration, and killing [65,87,88,146,147,148]. This mechanistic resolution is informative only when the relevant transport, barrier, and cellular functions are measured.

Flow, pressure, permeability, residence time, and gradient stability are biological variables rather than pump settings. Flow, pressure, vascular geometry, matrix stability under flow, and interstitial transport can alter exposure, invasion, transcription, or model performance [59,60,98,104,113,114,136,145]. Uniform perfusion may improve maintenance while erasing the heterogeneous delivery that the model is intended to reproduce.

Dynamic dosing and vascular barriers support transport-dependent pharmacology and evaluation of drug-delivery systems, but device materials, matrix properties, tubing/reservoir configuration, and fluidic residence time can alter free and delivered exposure [9,10,116,145,146,149]. Matrix-integrated tumour-on-chip systems are therefore particularly informative for questions involving penetration, barrier passage, spatial concentration, carrier transport, or exposure-dependent efficacy, provided the relevant transport variable is measured rather than inferred [10]. Loading patient tissue does not restore biology lost during processing. Published applications remain configuration-specific, and transfer requires qualification of tissue state, geometry, hydraulics, barrier function, compound or carrier recovery, and analysis [24,43,116].

### 4.6. Cross-Platform Integrated Multicellular Configurations

Integrated multicellular configurations combine tumour, stromal, vascular, immune, matrix, or transport modules when the endpoint depends on their coupling. They are not a distinct fabrication technology or platform class; they are cross-platform biological configurations that can be implemented in spheroids, organoids, matrix-engineered constructs, explants, or chips. Integration requires measurable cross-compartment transport, signalling, remodelling, recruitment, or response [59,65,113,114,116].

Perfused microvessels can change tumour maintenance and exposure, as shown by increased growth and heterogeneous drug delivery in a spheroid–microvascular system [59]. Sequential readouts can localize failure to vascular delivery, endothelial passage, matrix access, recognition, or intrinsic resistance [65,116]. Such resolution is the principal justification for integration; component count alone is not.

Each module also adds independent failure modes, including incompatible media, fibroblast overgrowth, vessel regression, immune dysfunction, and compounded pre-analytical variation. Integrated models should therefore be assembled and requalified modularly [24,43,116]. Evidence remains strongest for bespoke mechanistic studies of coupled transport, angiogenesis, immune trafficking, metastasis, or multicellular resistance; standardization, transferability, and clinical validation are less mature than component-level demonstrations.

### 4.7. Biology-Driven Platform Comparison and Model Selection

Biology-driven selection proceeds across the three orthogonal descriptors visualized in Figure 3 rather than by choosing from a single ranked list of platforms. Panel A compares five model formats by what they enable, the questions for which they are most useful, and their principal limitations: spheroids provide controlled three-dimensional organization and diffusion-dependent physiology [118]; tumour organoids provide expandable patient-derived tumour epithelium [122]; explants and slices provide short-term preservation of native tissue context [36]; matrix-engineered and bioprinted systems provide controlled matrix, mechanics, geometry, and cell placement [112]; and tumour-on-chip/MPS systems provide controlled flow, barriers, gradients, and compartmental communication [116]. These capabilities are complementary rather than hierarchical.

Panel B separates biological configuration from format. A tumour-only baseline, stromal, vascular, immune, metabolic-/gradient-controlled, or integrated multicellular configuration can be implemented in more than one format; the required function, rather than the platform label or component count, determines whether a configuration should be added. Tumour-cell screening may use spheroids or organoids; invasion may require a remodellable matrix; vascular delivery or immune recruitment requires a functionally qualified endothelial compartment; endogenous immune organization may favour short-term explants; and an integrated configuration is justified only when coupling among compartments changes the inference [36,59,65,106,116,118,122,126]. Uncertain functions should therefore be tested in matched modular configurations rather than assumed to improve physiological relevance.

Panel C separates implementation strategy from both format and configuration: preservation-oriented models retain the decision-relevant native state, reconstruction-oriented models rebuild selected functions after processing or de novo assembly, and hybrid models combine retained biology with engineered modules. Patient-specificity, physiological breadth, analytical control, and operational scale remain distinct properties. Organoids may preserve tumour-cell features while losing tissue context; explants retain context briefly with limited control; and engineered chips can reproduce transport without reproducing patient heterogeneity [36,116,122]. Throughput is likewise workflow-dependent—reflecting specimen requirements, culture duration, automation, dosing, imaging, and analysis—and should not be treated as an intrinsic property of a model format. Claims should remain limited to the functions and evidence actually demonstrated (Section 1.4; Supplementary Table S6).

### 4.8. Commercial Implementations: Architecture and Evidence Boundaries

Commercial products implement the architectures described in Section 3 and Section 4; they are not a separate biological class and do not constitute validated tumour models without assay-specific cells, matrices, transport conditions, exposure, endpoints, and controls. The brand-neutral main-text comparison is organized by generic architecture (Table 3). Provider-specific descriptions, peer-reviewed implementations, evidence provenance, and author-derived boundaries are separated in Supplementary Tables S5A and S5B.

Provider sources documented architecture, availability, compatibility, format, and stated use as rechecked on 4 September 2026. Official webpages are treated as time-stamped descriptive sources, not numbered items of scientific evidence; journal and conference-level oncology implementations are recorded separately with provenance [150,151,152,153,154,155,156,157,158,159]. A publication documents one implementation rather than every disease, configuration, predictive claim, or transfer pathway. Providers supplied no unpublished information, MatchCure products were excluded, and no product was ranked.

**Table 3:** Generic engineering architectures used in commercial tumour-model implementations. Architecture is a decision aid, not evidence of biological or predictive validation; provider-specific evidence is separated in Supplementary Tables S5A and S5B.

Architecture	Core Capability	Decision Question	Qualification Required
Perfused microfluidic well plate	Parallel 3D channels; gel patterning; perfusion; gradients	Do flow/gradients alter invasion, trafficking or pharmacology?	Cells/matrix/flow Delivered exposure and endpoint Parallelization ≠ validation [59,98,150]
Two-channel tissue-vascular chip	Separated tissue and vascular channels; controlled flow; barrier access	Does barrier transport or vascular signalling alter exposure or entry?	Lumen/barrier function Permeability and delivered exposure Loading ≠ restoration of lost biology [65,146,151]
Hydrogel chamber + perfusion channels	Spatial compartments; gradients; migration interfaces	How do invasion, angiogenesis, extravasation or immune migration occur?	Cell/gel quality Flow/pressure and migration route Compound recovery [88,114,152]
Scalable spheroid/microtissue plate	Controlled aggregation; microplate dosing/imaging	Does activity persist in 3D or defined co-culture at scale?	Size/gradients/internal viability Assay dynamic range No inherent circulation [70,118,119,120,121,153]
Recirculating multi-compartment MPS	Linked tissue chambers; dynamic medium recirculation	How do inter-tissue signalling, metabolism and efficacy-toxicity interact?	Inter-tissue communication Exposure; not organism-level PK [154,156,160,161]
Millifluidic/open-channel chamber	Accessible tissue loading under dynamic flow	How does tissue respond under dynamic exposure or immune infiltration?	Transport and throughput Evidence is implementation-specific [155,157,158]

Abbreviations: 3D, three-dimensional; MPS, microphysiological system.

## 5. Applications in Drug Discovery and Functional Precision Oncology

Applications are considered only when a model resolves a decision-relevant uncertainty: whether activity persists under tissue constraints, which mechanism causes resistance, whether a therapeutic can reach the relevant compartment at an adequate exposure, whether immune cells can reach and eliminate tumour targets, or whether an ex vivo response is associated with a treatment-specific clinical outcome. This makes the framework directly relevant to pharmaceutics: small molecules, biologics, nanomedicines, and cell- or gene-based therapies may fail because of pharmacodynamic insensitivity, delivery barriers, exposure loss, or treatment schedule, and these mechanisms require different model functions and readouts [10]. The intended decision, decisive biological function, exposure model, and actionable endpoint should therefore be defined before platform selection.

### 5.1. Drug Discovery and Candidate Prioritization

Drug discovery benefits from a tiered strategy. Two-dimensional systems remain appropriate for high-throughput hit identification, target validation, and structure–activity work, whereas multicellular models are most useful as secondary or tertiary filters when tissue state, matrix, stroma, transport, or immune access may reorder candidate performance [2,162,163].

Platform choice should follow therapeutic mechanism. Spheroids can test growth-state-dependence and penetration; organoid panels can examine interpatient variation; paired tumour–normal systems can examine selectivity; and perfusion-based systems can examine delivery-dependent effects [160,162,163]. Organoid screens have been used to identify therapeutic candidates and context-specific vulnerabilities [162,163,164], while single-organoid screening technologies can increase analytical resolution within heterogeneous organoid populations [165].

Exposure realism is cross-cutting. Nominal medium concentration may differ from free and delivered exposure because of protein or matrix binding, metabolism, chemical instability, device or tubing adsorption, barrier transport, diffusion distance, cellular uptake, and clearance or washout. Dosing schedule, infusion or bolus approximation, repeated exposure, washout, recovery, and clinically achievable exposure should therefore be justified across spheroids, organoids, explants, matrices, and chips [9,10].

For drug-delivery systems, a useful model should measure the transport step that the formulation is intended to change. Depending on the product, this may include carrier stability, matrix partitioning, barrier passage, tissue penetration, spatial concentration, release kinetics, cellular uptake, residence time, and recovery of intact drug or carrier. A decrease in viability after nominally identical dosing cannot distinguish improved delivery from altered cellular sensitivity unless exposure is quantified. Matrix-integrated microfluidic tumour models are especially suited to such questions because transport and tissue response can be measured within the same configuration [10].

Candidate ranking should combine longitudinal growth, apoptosis, spatial viability, and regrowth rather than rely on one ATP or metabolic endpoint. Matrix-source changes can alter organoid growth without materially shifting dose–response in the same configuration [166], illustrating that added biological detail does not necessarily improve ranking. Added complexity is useful only when it changes ranking or explains discordance through proliferation, stromal protection, immune access, or delivery.

### 5.2. Mechanism-of-Action and Therapeutic Resistance Studies

Mechanism-of-action studies require controlled perturbation, whereas resistance studies additionally require preservation of adaptation over time. Defined systems can isolate matrix stiffness [106], fibroblast signalling [97], endothelial transport [59], immune-cell-mediated cytotoxicity [64], or metabolic stress [93]. Causal evidence requires perturbing the proposed mechanism and changing the treatment phenotype; a response difference between model formats is not sufficient by itself.

Multicellular models are particularly valuable for non-cell-autonomous resistance. Stromal cells can alter response through metabolite support and paracrine survival signalling [28,52]; tumour-associated immune and myeloid states can create suppressive microenvironments [63,83]; perfusion and vascular geometry can alter delivered drug exposure [59,60]; and nutrient competition can impair antitumour T-cell metabolism [69]. Matched tumour-only and multicellular conditions, followed by selective perturbation of the implicated compartment, are needed to distinguish these effects from tumour-intrinsic resistance.

Resistance is temporal and treatment-specific. Short endpoint assays can miss delayed death, recovery, lineage switching, antigen loss, and expansion of drug-tolerant populations; longitudinal imaging, repeated sampling, and post-treatment regrowth are therefore important [167,168,169,170,171]. Patient treatment history can also be captured unevenly: metastatic colorectal organoids retained oxaliplatin resistance, whereas acquired resistance to fluorouracil and irinotecan was only partly reproduced [172]. This discordance shows that preservation of resistance cannot be assumed across drugs, even within the same platform.

Combination testing should be hypothesis-driven and reproduce the targeted compartment and exposure sequence. Antiangiogenic combinations may depend on a transient vascular-normalization window [57,58], whereas stroma- and immune-directed combinations require a functionally competent stromal or immune compartment [52,101,102]. These models are strongest when matched perturbations discriminate a defined resistance mechanism; broad claims of ‘resistance modelling’ remain unsupported when adaptation, exposure, and recovery are not measured.

### 5.3. Immuno-Oncology and Cellular Therapy Evaluation

Immuno-oncology models should reproduce the therapeutic step under study. Simple direct tumour–lymphocyte co-culture can assess recognition and killing but does not by itself establish recruitment, endothelial crossing, matrix infiltration, antigen presentation, or myeloid suppression [64,86,87,173]. Vascularized immune systems can resolve tissue entry [65,88], whereas explants and air–liquid-interface cultures can preserve endogenous immune and stromal organization for short assay windows [36,37,103,126,133]. Checkpoint therapy and engineered-cell therapy therefore require different cell sources, routes of introduction, controls, and stage-resolved endpoints.

Patient-derived organoid–immune co-cultures offer experimental control, but validity depends on immune-cell source, HLA compatibility, activation state, culture medium, tumour antigenicity, and assay duration. Organoid culture conditions can suppress lymphocyte function [64]. Allogeneic systems therefore require explicit controls for non-tumour-specific recognition. Autologous systems preserve patient compatibility but are constrained by cell yield, expansion time, and phenotypic drift. Preservation-oriented models may better support acute checkpoint-associated studies, but progressive immune loss and ex vivo adaptation must be measured rather than assumed to be absent.

Final tumour viability is an incomplete endpoint for cellular therapies. BEHAV3D resolved migration, contact behaviour, and killing states among engineered T cells [86], while vascularized tumour chips required adhesion under flow, endothelial crossing, matrix migration, tumour engagement, and cytotoxicity and could also reveal endothelial injury [65]. Such stage-resolved measurements distinguish failure of delivery from failure of recognition or effector function and provide more actionable engineering information than a terminal viability value.

Current evidence supports immune-integrated models mainly as mechanistic and comparative pharmacological tools. Sample-specific or stroma-associated responses have been demonstrated [88,101,102,103], but these systems omit systemic priming, lymphoid-organ interactions, organism-level pharmacokinetics, and immune-related toxicity. Patient derivation and ex vivo response should therefore not be described as prospective predictive validity unless the assay configuration and analysis rule are locked before evaluation in an independent treatment-specific validation cohort; clinical utility additionally requires evidence that returning the result improves a decision or outcome.

### 5.4. Functional Precision Oncology

Translation should distinguish retrospective association, prospective observational evaluation, prospective blinded prediction, assay-guided allocation, and clinical utility. Only the last tests whether returning a result through a predefined rule changes management and improves a clinically relevant outcome or decision; feasibility, turnaround, and assay success remain necessary but separate operational properties.

Representative studies differ in specimen, assay, regimen, threshold, denominator, and outcome definition (Supplementary Tables S4A and S4B). Evidence spans treatment-specific external association and prospective observational outcome-association or predictive-performance analyses across organoid workflows [127,128,130,131,174,175], together with a prospective blinded predictive-performance analysis in ovarian-cancer spheroids [176]. These descriptors summarize how performance was assessed; they do not constitute prospective predictive validity when the assay rule or threshold was developed, confirmed, or retuned within the evaluated cohort. Differences across colorectal regimens and evolving workflow performance illustrate dependence on cohort, assay maturity, endpoint, and analysis rule [127,130]. A fully 3D-printed tumour-on-chip study also reported a correlation between ex vivo drug response and clinical outcomes, supporting limited external association rather than prospective predictive validity [177].

Recurrent limitations include assay failure, conditioning on specimen adequacy, selected evaluable cohorts, internally derived thresholds, treatment chosen independently of the assay, treatment availability, prior therapy, sampling heterogeneity, and delayed reporting [127,130,131,174,175,176]. Full eligible denominators and failure reasons are therefore part of performance assessment. None of the representative studies establishes clinical utility.

Prospective validation should predefine eligibility, specimen and exposure requirements, response metric, reporting window, threshold, treatment-specific analysis, and clinical endpoint [127,130,131,174,175,176]. Predictive-validity claims require blinding and a locked assay configuration, analysis pipeline, endpoint, and prediction rule evaluated without retuning in an independent validation cohort. Assay-guided studies should document whether the result changed management, whether the recommended treatment was delivered, and how outcomes compare with a prespecified alternative strategy. Until utility is established for a defined assay–disease–treatment combination, functional results should remain complementary evidence rather than validated off-label treatment guidance. Table 4 links four application classes to their decisive function, priority readouts, and principal interpretive boundary. Platform selection is addressed in Table 2, and comparative oncology is considered separately as an external-validation environment in Section 6.5.

## 6. Challenges, Validation, and Future Directions

Translation requires three linked forms of control: biological relevance, analytical and operational qualification, and validation for a defined context of use. Complexity without qualified function produces ambiguous systems, whereas standardization that removes decisive biology can produce reproducible but uninformative assays [24,25,43].

### 6.1. Biological Fidelity Versus Experimental Complexity

Biological fidelity, analytical robustness, predictive validity, and clinical utility are distinct. Complexity may arise from biological compartments, engineered transport, operations, readouts, or computation. The relevant question is whether the function governing the endpoint is present and measured reliably enough for the proposed decision.

The author-proposed minimal-sufficient-model principle is conditional on whether the study is mechanism-guided or discovery-oriented. In mechanism-guided work, nested configurations test whether a prespecified module is required for the inference. In discovery-oriented work, controlled complexity is itself an experimental variable: several plausible modules may initially be retained so that the determinant of the phenotype can be localized. Only after that determinant is identified and perturbed should the configuration be reduced to the minimal explanatory set. Consistent with fit-for-purpose qualification principles [24,43], a module is retained when a prespecified comparison reproducibly changes mechanism, response ranking, uncertainty, or decision-relevant interpretation, and excluded when burden or ambiguity increases without reducing the uncertainty that motivated its inclusion. 

### 6.2. Reproducibility, Standardization, and Quality Control

Reproducibility has pre-analytical, technical, biological, pharmacological, and computational components. Specimen handling, processing, passage, cryopreservation, exposure, and analysis can all alter results [36,37,38,39,40]. Eligibility, failures, delivered exposure, analysis version, and turnaround should therefore be recorded, while standardization should reduce avoidable noise without suppressing intended biological heterogeneity [24,25,43].

Quality criteria must match model class and context of use. Spheroids require establishment, size, viability, internal state, and dynamic range [70,118,120]; organoids additionally require identity, establishment denominator, passage, matrix/medium, and control of normal-cell overgrowth [122,123,126]. Explants require tumour content, injury, exposure route, and assay-window stability [36,37,132,134], while vascular and immune models require direct qualification of transport or immune function [59,64,65,178].

Controls should test the claimed function and its principal alternative explanation. Endothelial systems may require no-flow, permeability, or barrier-perturbation controls; immunotherapy assays require target-positive/-negative comparators, alloreactivity assessment where relevant, and functional blockade [59,64,65]. Acceptance ranges, missing-data rules, calibration, and failure criteria should distinguish technical failure from biological variation [24,25,43].

Interlaboratory reproducibility remains constrained by incomplete reporting of matrix, processing, device, flow, medium, exposure, and analysis [24,43,178]. Shared reference materials, standardized reporting, and transfer studies are priorities [24,179]. Table 5 summarizes the main model-class requirements; the expanded checklist is provided in Supplementary Table S11.

### 6.3. Context-of-Use and Performance Validation

Figure 4 operationalizes the validation framework as a non-cumulative two-track evidence matrix anchored to the context of use and intended claim. Track A asks what has been demonstrated about the model itself and separates identity/structural fidelity, functional fidelity, and comparative pharmacological response evidence. Track B asks what external evidence supports the intended claim or decision and separates external association, prospective predictive validity, and clinical utility. The six domains are independently assignable: a study may satisfy any subset supported by its data, and the presence of one domain does not establish another. Analytical/operational qualification—repeatability, controls, pre-analytical robustness, delivered exposure or recovery where relevant, endpoint stability, assay failure/denominator reporting, and workflow/version control—supports interpretation across both tracks rather than constituting an additional stage [24,25,43].

For study classification, ‘predictive-performance evidence’ is used only as a descriptive modifier of external association when an assay is compared with treatment-specific outcomes. It is not a separate validation level and is not synonymous with prospective predictive validity. If an assay threshold, score, or analysis rule is developed, optimized, or confirmed using the same cohort in which performance is evaluated, the study remains in the external-association domain regardless of prospective enrolment or blinding.

Analytical validation should characterize repeatability, intermediate precision, pre-analytical sensitivity, control separation, calibration, robustness, failure, delivered exposure, endpoint stability, missingness, and change control [24,25,43]. Functional claims require function-specific measurement or perturbation; comparative pharmacological response additionally requires a justified comparator, defined exposure, prespecified endpoints, and reproducible analysis [59,64,65]. External validation must use a benchmark matched to the claim, with full denominators, failures, treatments, missing outcomes, and uncertainty reported for predictive studies [127,130,131,174,175,176].

Orthogonal endpoints should resolve plausible alternatives: transport measures distinguish delivery failure from tumour-cell insensitivity [59], immune trajectories separate tissue access from recognition or killing [65], longitudinal recovery distinguishes transient cytostasis from durable control, and analytical controls separate biological variation from assay failure [24,43]. Table 6 summarizes the evidence domains and their claim boundaries.

### 6.4. Validation of the Proposed Decision Framework

The two-entry decision sequence, function classification, biology-to-decision record, orthogonal taxonomy, and minimal-sufficient-model rule proposed in this review have not been prospectively or independently validated. The worked examples are explanatory applications of the framework, not evidence that framework-guided selection improves experimental or translational decisions.

Framework-level validation should use predefined mechanism-guided and discovery-oriented scenarios evaluated independently by multidisciplinary users. Outcomes should include agreement in defining the decision, classifying mechanism status and candidate functions, selecting the format/configuration/strategy, choosing functional controls, applying the complexity-retention rule, and recognizing unsupported claims. The completed biology-to-decision record provides an auditable unit for comparing framework-guided decisions with conventional platform-centred selection.

Prospective evaluation should then use nested or factorial model series to test whether framework-selected retained or excluded modules change the mechanism, response ranking, uncertainty, or interpretation. Independent transfer should assess reproducibility, time, cost, failure rate, and decision-relevant information gain. A particularly testable prediction is that the framework will sometimes justify de-escalation to a simpler configuration and in other cases justify targeted escalation; superiority should not be assumed in advance. Until these decision rules are prospectively evaluated across laboratories, the framework remains a transparent decision aid rather than a validated standard.

### 6.5. Comparative Oncology as an External Validation Environment

Comparative oncology is an external-validation environment, not a separate in vitro platform class. Naturally occurring companion-animal cancers can be used to examine conserved mechanisms, tissue-level constraints, pharmacological response, or functional-assay signals in intact disease. Veterinary functional testing is a related clinical application but should be distinguished from cross-species mechanistic validation and from prediction of human treatment response.

Naturally occurring companion-animal cancers arise in immunocompetent hosts; recent canine studies have examined targeted agents, immunotherapy, local intervention, and spatial biology [180,181,182,183,184]. Veterinary organoids and other patient-derived models can generate comparative and, in selected reports, paired model–clinical datasets [185,186,187,188,189]; preliminary abstract-level evidence is also emerging [190].

These systems are not automatically superior external benchmarks. Interpretation requires explicit correspondence in pathology and mechanism, and standardized tumour assessment is important for reproducible veterinary comparisons [191]. Species, breed, tumour incidence, pharmacology, standards of care, assay success, treatment allocation, and outcome definitions remain additional context-specific considerations.

Evidence strength depends on the claim and design. Conserved mechanisms may support external mechanistic relevance; predefined ex vivo–clinical comparisons may support external association or prospective predictive validity within veterinary care; and veterinary clinical utility would require an assay-guided decision rule with comparative outcome evidence. None of these categories substitutes for human validation. Comparative oncology should therefore test a specific cross-species or veterinary claim rather than serve as a rhetorical bridge to human benefit.

### 6.6. Scale, Automation, Manufacturability, and Data Integration

Scalability means controlled production, operation, and analysis at the throughput required by the context of use, not simply more wells. Robotic hydrogel fabrication, high-throughput bioprinting, three-dimensional tumour–T-cell-array analysis, organoid-array computation, high-content imaging, and cell tracking illustrate different forms of scale [192,193,194,195,196,197]. Automation requires calibration, process controls, acceptance criteria, exception handling, and traceable specimen-to-result metadata [24,43].

Manufacturability becomes harder with primary cells, multiple media, biological matrices, and perfused devices. Material variability, device fabrication, reagent handling, instrument-dependence, and operator intervention can limit transfer [24,25,178,192,193]. Stability should be assessed using the function and endpoint used for decision-making, not morphology alone, and changes to materials or workflows should trigger risk-based requalification.

Multidimensional imaging and computational analysis can quantify spatial organization, vessels, immune behaviour, and temporal response. Recent computational platforms illustrate machine-learning-guided regimen search, high-content single-cell image analysis, and cell tracking with explicit confidence estimation [195,196,197]. For translational use, analysis workflows should additionally predefine reference annotations, training/validation separation, calibration or error estimation, version control, and handling of low-confidence outputs. Shared metadata conventions and standardized protocols should link specimen, configuration, exposure, analysis, and outcome while preserving provenance and traceability [24,25,179].

### 6.7. Regulatory and Translational Adoption

Regulatory interest in New Approach Methodologies has expanded, but acceptance remains context-specific and weight-of-evidence based. Recent FDA experience emphasizes use of NAMs within defined drug-development questions rather than as blanket replacements for existing evidence streams [23]. OECD good in vitro practice guidance and organ-on-chip design and operation guidance likewise emphasize fit-for-purpose design, documentation, and reproducible operation [25,179]. Scientific acceptance, use as supportive evidence, qualification for a specific context of use, and replacement of an existing test are therefore distinct outcomes.

Fit-for-purpose development links model design and operation to the intended application, while regulatory qualification additionally requires alignment of the endpoint, operating range, decision rule, and regulatory question [23,25,179]. Standard procedures, version-controlled materials and software, predefined acceptance criteria, data integrity, failure reporting, and change control are essential. Changes to cells, matrix, geometry, flow, exposure, analysis, or reporting may require revalidation when they affect the qualified function or decision boundary.

Clinical adoption additionally requires reliable tissue access, pathology coordination, consent and governance, timely reporting, clinically interpretable outputs, and integration with established evidence. Reports should state assay success, exposure ranges, uncertainty, mechanism coverage, and unsupported treatment classes. Regulatory acceptance does not establish clinical utility; until prospective utility is shown, functional results should complement rather than replace pathology, molecular diagnostics, pharmacology, and clinical judgement [131,174,175,176].

### 6.8. Next-Generation Tumour Models

Next-generation tumour models should become more selective, measurable, transferable, and interoperable—not simply more elaborate. First, modular platforms should add context-specific components only when matched comparisons show decision-relevant information gain. We propose treating disease- or organ-specific vascular, stromal, neural, endocrine, metabolic, or metastatic-niche functions as explicit modules with their own variables and acceptance criteria rather than assuming that they emerge from generic complexity.

Second, models of adaptation should capture time through sequential assembly, clinically informed treatment schedules, repeated exposure, longitudinal imaging, washout, regrowth, and non-destructive sensing [94,168,170,171,195]. Spatial and single-cell profiling can benchmark treatment-associated or culture-induced states, but correspondence with parental or longitudinal tissue should be tested rather than assumed [15,16,30,39,95]. Third, tumour models should be evaluated with relevant normal tissues when therapeutic selectivity, barrier injury, or toxicity is decisive. Parallel tumour–normal organoids and linked tumour–vascular or multisensor systems illustrate this approach [94,160,161] but require validated exposure, inter-tissue communication, normal-tissue endpoints, and evidence that linking compartments improves the intended decision. 

Fourth, computation should guide experimental design, quantify images, integrate modalities, and express uncertainty rather than conceal it [95,195,196,197]. Digital tumour twins remain aspirational; clinically useful counterparts would require prospectively validated links among patient data, model behaviour, delivered exposure, treatment decisions, and longitudinal outcome.

Priorities include broader patient representation and comparative systems, more defined matrices, transferable fabrication, shared benchmarks, and transparent failure reporting [24,25,104,185,191]. Generalization should be demonstrated across specimen sources, laboratories, operators, instruments, and analysis versions rather than inferred from technical sophistication [24,25,43]. Figure 5 consolidates the framework into a practical selection aid: begin with the scientific, pharmacological, or translational decision; choose a mechanism-guided or discovery-guided entry route; specify and qualify the model; evaluate added complexity using a three-outcome rule—retain when a decision-relevant effect is demonstrated, simplify only when a meaningful effect is adequately excluded, and classify the result as inconclusive when precision or sensitivity is insufficient—and then match the demonstrated evidence to a supported, bounded claim. The workflow deliberately separates model qualification from external association, prospective predictive validity, and clinical utility and should not be read as a ladder of increasing model complexity or evidentiary maturity. 

## 7. Conclusions

Physiological relevance depends on reproducing the functions that govern a defined decision, not on three-dimensional form, patient origin, or maximal complexity. The proposed biology-driven framework therefore begins with the scientific, pharmacological, or translational decision and uses two entry routes. Mechanism-guided studies can prespecify the candidate causal function and begin with a minimal-sufficient configuration; discovery-oriented studies instead treat plausible functions and model modules as experimental variables and derive a minimal explanatory configuration only after the determinant of the phenotype has been localized and perturbed. Model format, biological configuration, and implementation strategy should be described separately because an integrated multicellular configuration can be implemented across several technological formats.

Within the representative evidence appraised in this review, identity/structural fidelity, functional fidelity, and configuration-dependent pharmacological response were documented more often than independently validated prospective predictive validity or clinical utility. Patient-derived origin cannot compensate for missing decision-critical biology, and added components do not establish function without measured information gain. For pharmaceutical development, the nominal dose should likewise not substitute for qualified exposure: matrix or protein binding, barrier transport, device loss, tissue penetration, metabolism, release, and dosing schedule should be measured when they can alter efficacy, resistance, or delivery conclusions. The practical contribution of the framework is an auditable biology-to-decision record that links mechanism status, candidate functions, format/configuration/strategy, qualification evidence, complexity-retention rules, and claim boundaries. Its value should be tested prospectively by asking whether it changes experimental design, improves recognition of unsupported claims, and yields decision-relevant information with acceptable reproducibility and burden. Until such validation is completed, it remains an author-proposed decision aid rather than a validated standard.

## Figures and Tables

**Figure 1:**
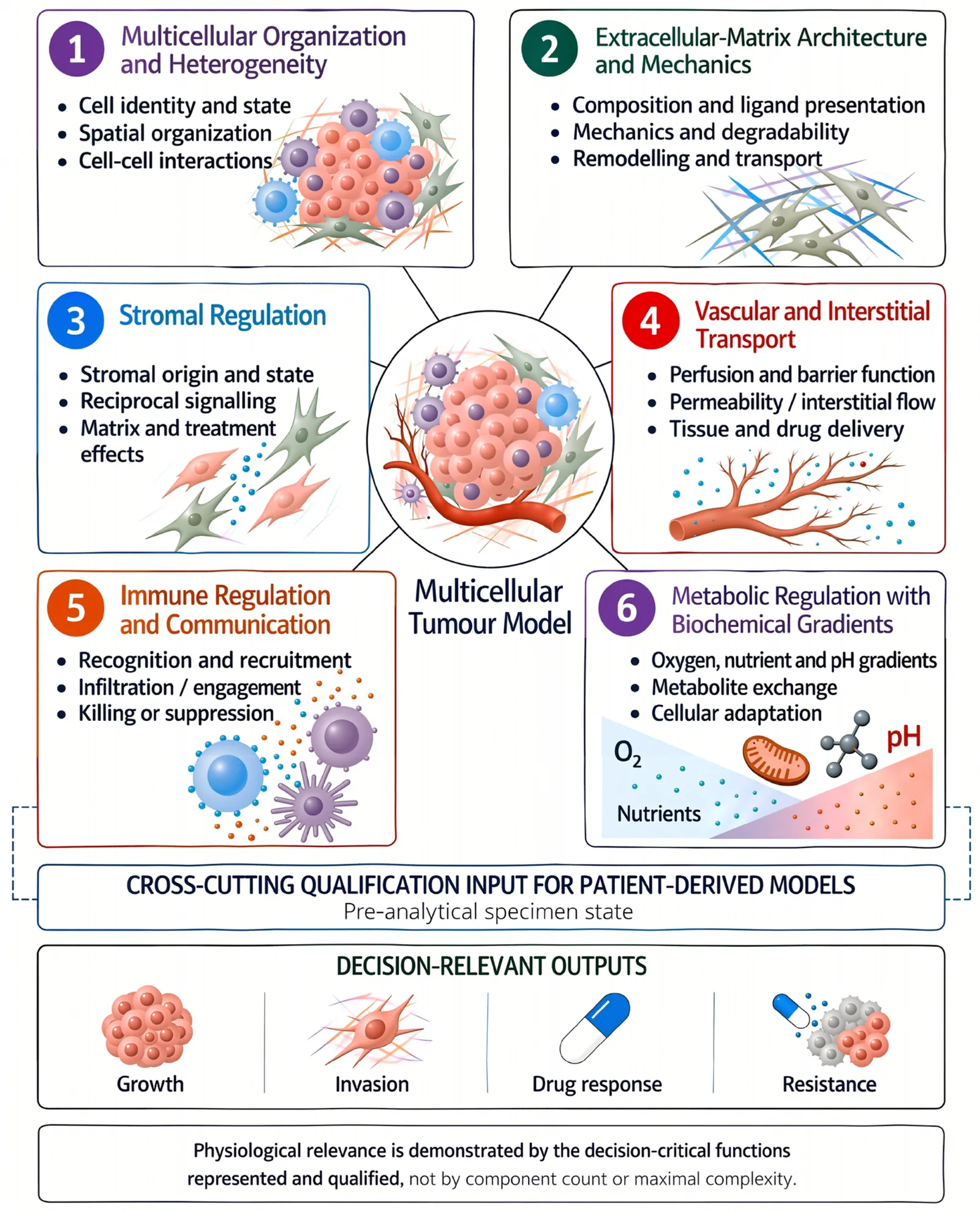
Biological determinants and cross-cutting qualification inputs for physiologically relevant multicellular tumour models. The central multicellular tumour model is linked to six coupled biological domains: (1) multicellular organization and heterogeneity; (2) extracellular-matrix architecture and mechanics; (3) stromal regulation; (4) vascular and interstitial transport; (5) immune regulation and communication; and (6) metabolic regulation with biochemical gradients. For patient-derived models, pre-analytical specimen state is shown as a cross-cutting qualification input rather than a seventh biological domain. Growth, invasion, drug response, and resistance are shown as illustrative decision-relevant outputs through which the required functions may be evaluated; these outputs are not exhaustive and do not by themselves establish predictive validity or clinical utility. Physiological relevance is therefore demonstrated by the decision-critical functions represented and qualified, not by component count or maximal complexity. Abbreviation: ECM, extracellular matrix.

**Figure 2:**
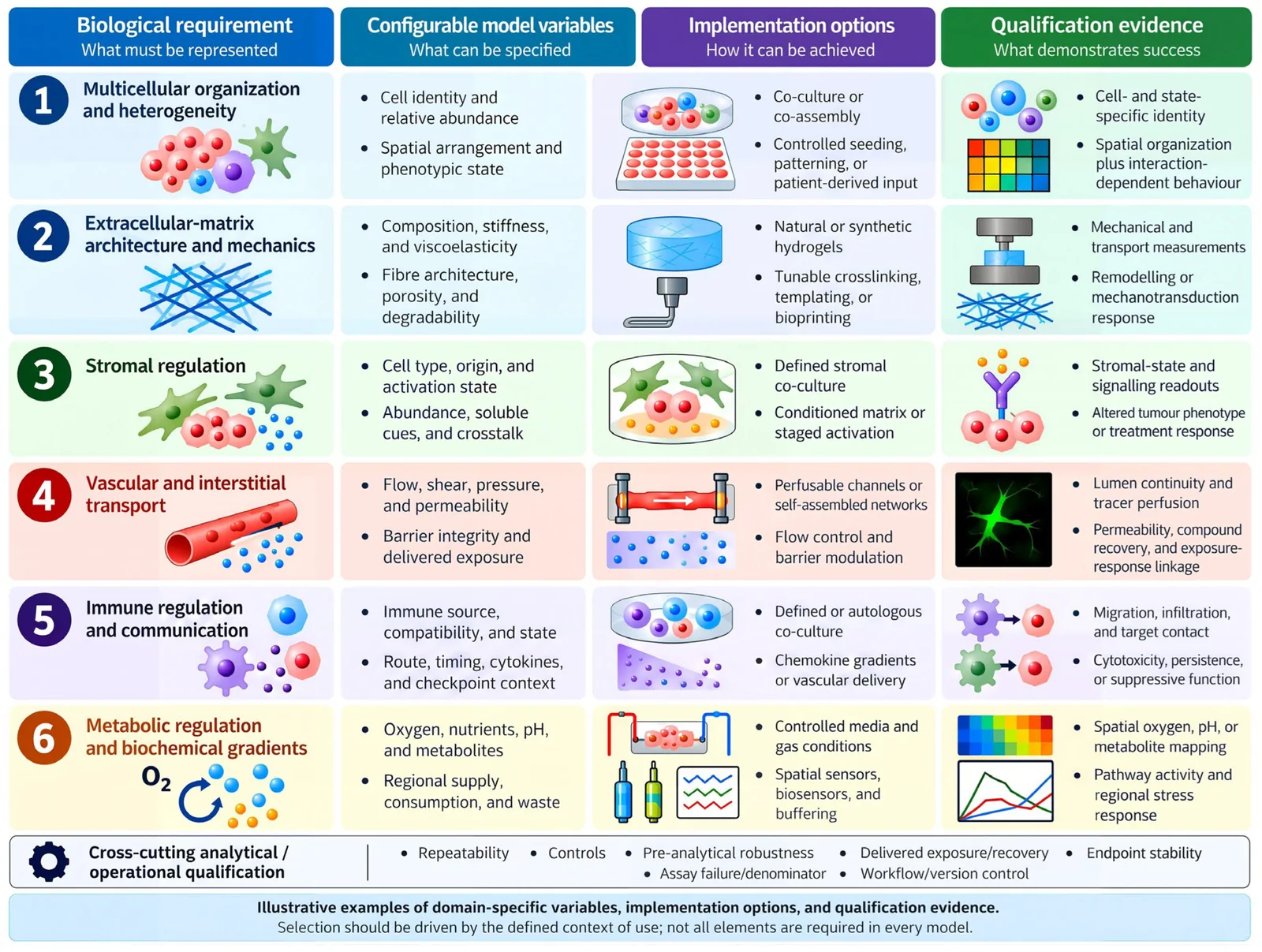
Biology-to-engineering translation matrix for the six decision-critical biological domains. Each row translates one biological requirement—multicellular organization and heterogeneity, extracellular-matrix architecture and mechanics, stromal regulation, vascular and interstitial transport, immune regulation and communication, or metabolic regulation with biochemical gradients—into three design layers: configurable model variables, implementation options, and qualification evidence. The examples illustrate how cell composition and spatial state, matrix properties, stromal state, flow and barrier properties, immune context, and biochemical conditions can be specified and implemented and how the corresponding structure or function can be measured directly. Analytical/operational qualification is cross-cutting across all domains and includes repeatability, controls, pre-analytical robustness, delivered exposure or recovery where relevant, endpoint stability, assay-failure/denominator reporting, and workflow/version control. The matrix is illustrative rather than prescriptive: not all variables, implementation options, or qualification measurements are required in every model, and selection should be driven by the defined context of use.

**Figure 3:**
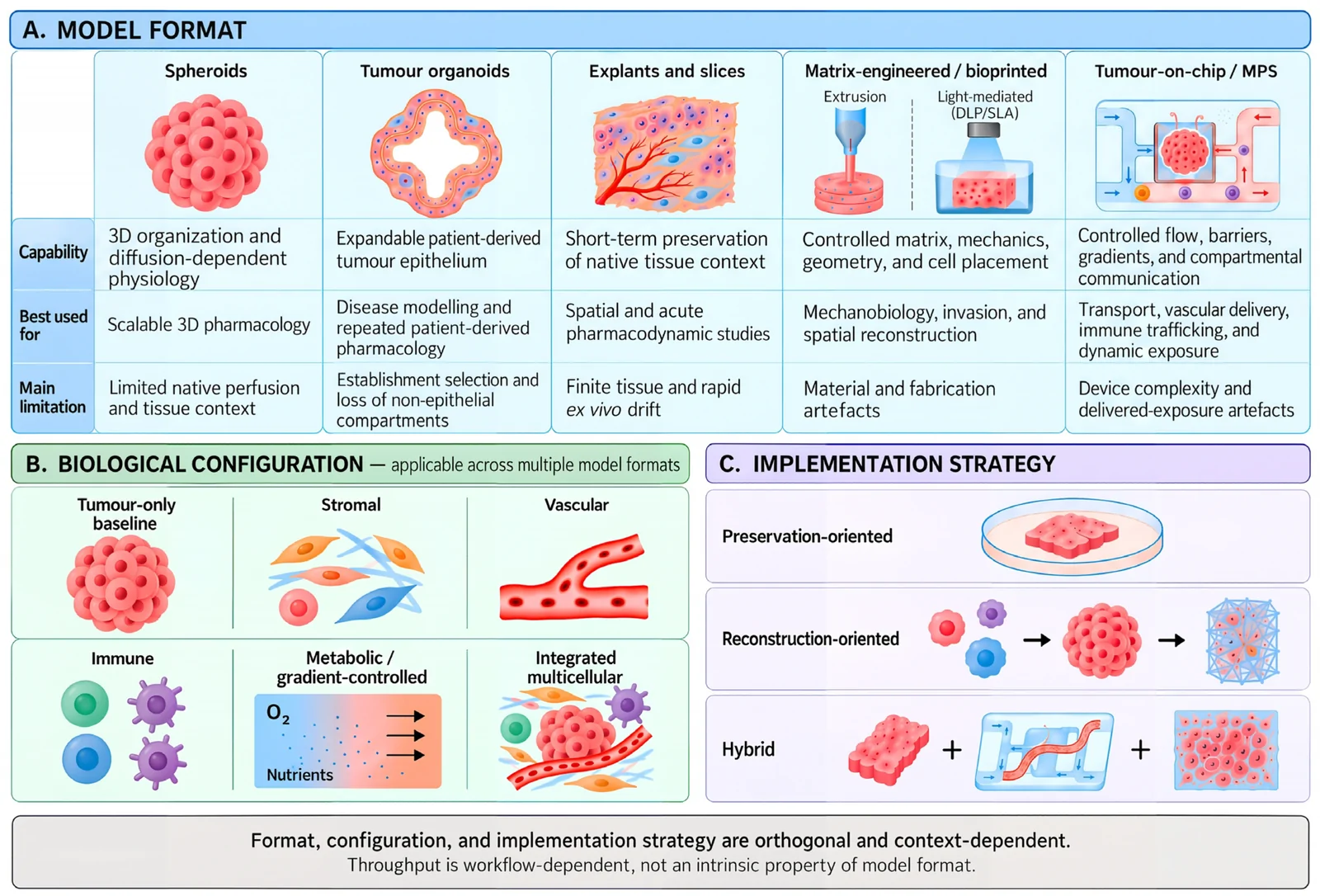
Orthogonal classification of multicellular tumour-model formats, biological configurations, and implementation strategies. Panel A compares five model formats—spheroids, tumour organoids, explants and slices, matrix-engineered/bioprinted models, and tumour-on-chip/MPS—using concise capability, best-use, and principal-limitation descriptors. Extrusion-based and light-mediated (DLP/SLA) fabrication are shown as distinct implementation approaches within the matrix-engineered/bioprinted format; neither is implied to be intrinsically more physiological. Panel B shows cross-cutting biological configurations that can be implemented across multiple formats: tumour-only baseline, stromal, vascular, immune, metabolic-/gradient-controlled, and integrated multicellular. Integrated multicellular is therefore a biological configuration rather than a separate technological platform class. Panel C distinguishes preservation-oriented, reconstruction-oriented, and hybrid implementation strategies as a separate axis. The three dimensions are orthogonal and context-dependent, and no format or configuration is presented as universally superior. Throughput is workflow-dependent rather than an intrinsic property of model format. Abbreviations: DLP, digital light processing; MPS, microphysiological system; SLA, stereolithography.

**Figure 4:**
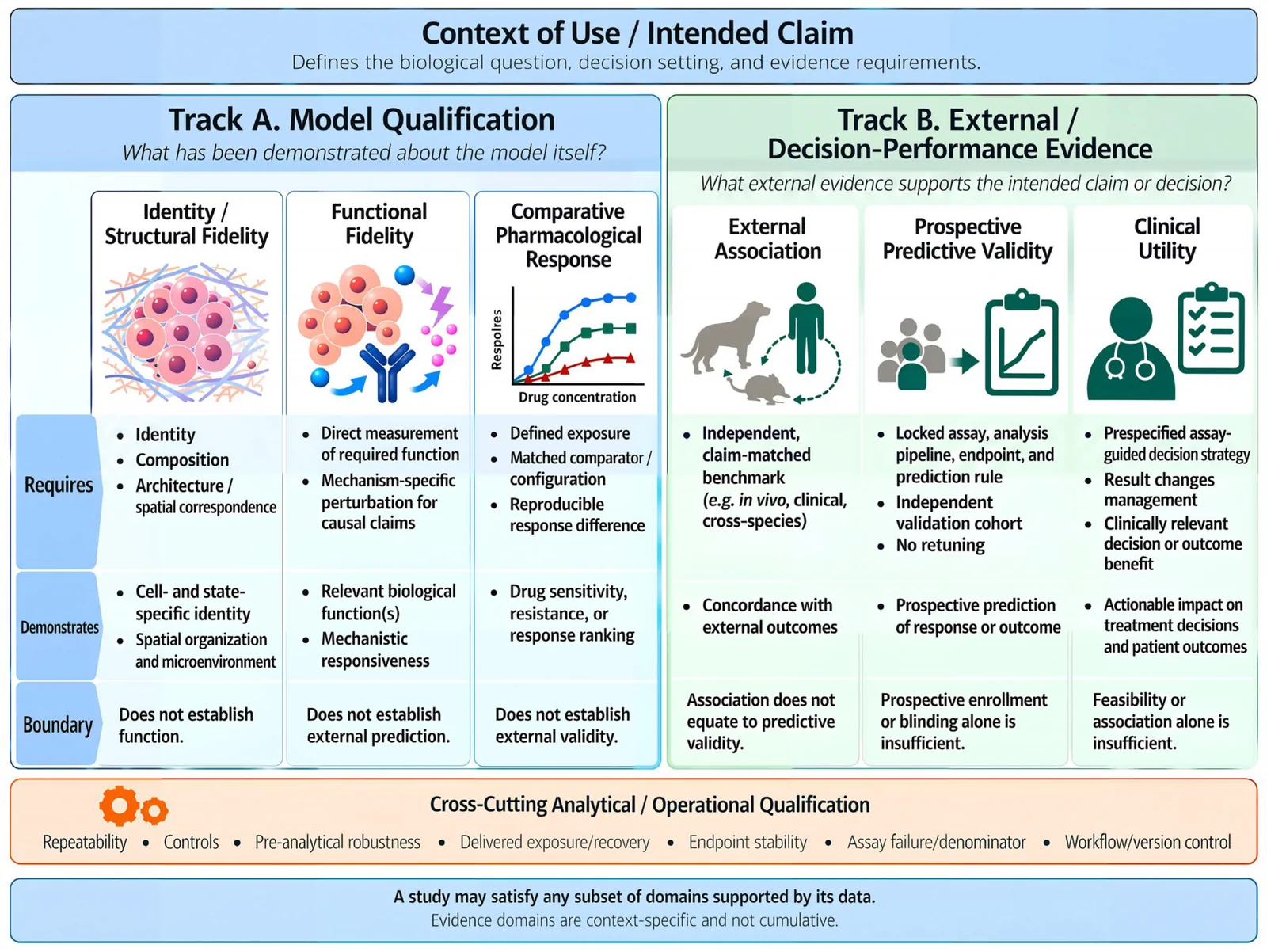
Non-cumulative evidence framework for model qualification and claim validation. The defined context of use and intended claim determine which evidence domains are relevant. Track A comprises three independently assignable model-qualification domains: identity/structural fidelity, functional fidelity, and comparative pharmacological response. Track B comprises three independently assignable external or decision-performance domains: external association, prospective predictive validity, and clinical utility. Identity/structural fidelity does not establish function; functional fidelity does not establish external prediction; comparative pharmacological response does not establish external validity; external association does not equate to predictive validity; prospective enrolment or blinding alone is insufficient for prospective predictive validity; and feasibility or association alone is insufficient for clinical utility. Prospective predictive validity requires a locked assay, analysis pipeline, endpoint, and prediction rule evaluated without retuning in an independent validation cohort. Clinical utility requires a prespecified assay-guided decision strategy, evidence that the result changes management, and a clinically relevant decision or outcome benefit. Analytical/operational qualification is cross-cutting across both tracks. A study may therefore satisfy any subset of evidence domains supported by its data; the domains are context-specific and not cumulative.

**Figure 5:**
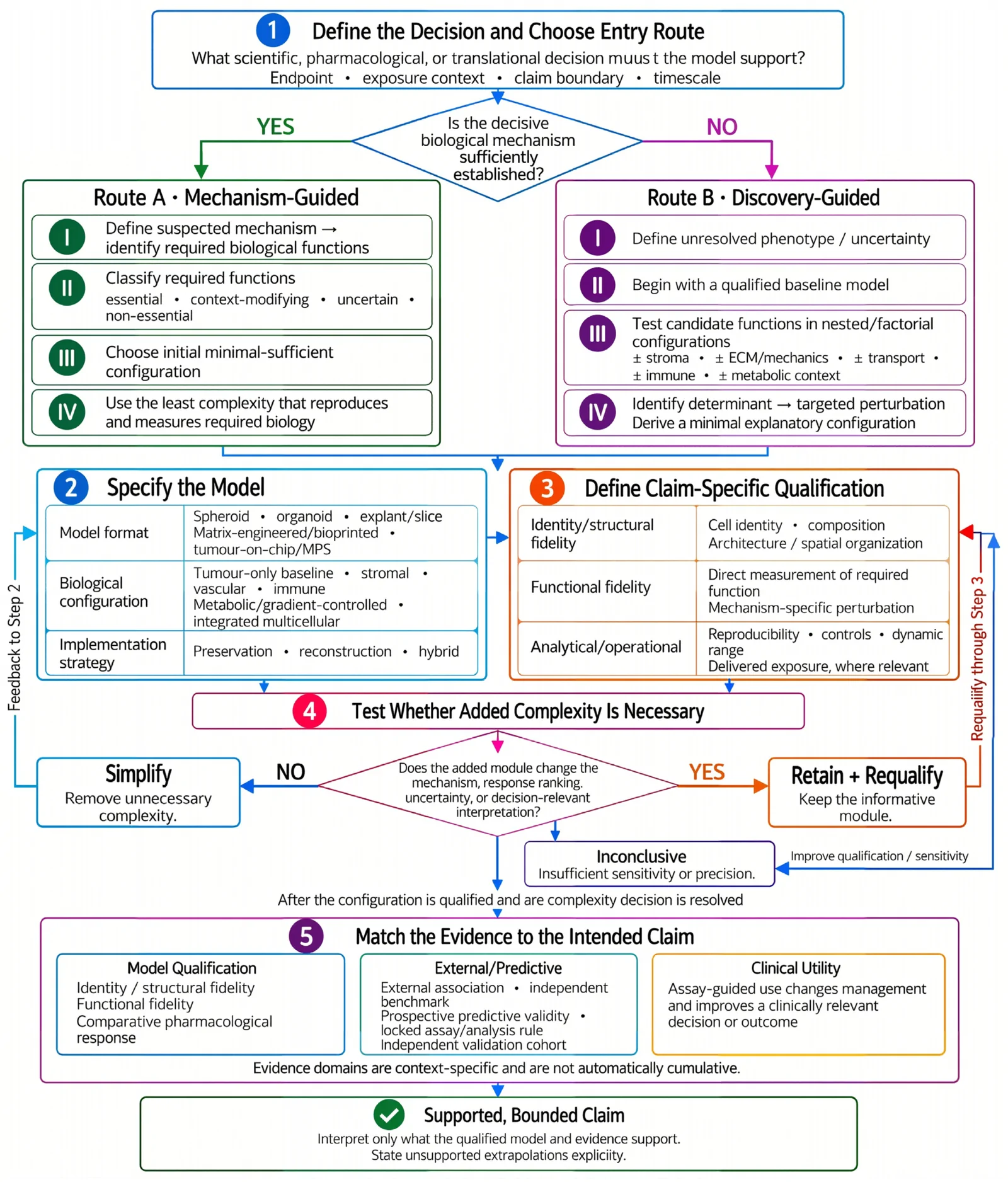
Biology-driven decision workflow for selecting and qualifying multicellular tumour models. Step 1 defines the scientific, pharmacological, or translational decision and selects a mechanism-guided or discovery-guided entry route. In the mechanism-guided route, required biological functions are specified from a plausible decisive mechanism and used to choose an initial minimal-sufficient configuration. In the discovery-guided route, unresolved candidate functions are tested through nested or factorial configurations, the determinant is localized and perturbed, and a minimal explanatory configuration is derived. Both routes then converge on Step 2, specification of the model using three orthogonal descriptors—model format, biological configuration, and implementation strategy—and Step 3, claim-specific identity/structural, functional, and analytical/operational qualification. Step 4 applies a three-outcome complexity test. An informative module is retained and requalified when a reproducible decision-relevant effect is demonstrated; the configuration is simplified only when a qualified comparison has sufficient precision and sensitivity to exclude a prespecified decision-relevant effect; and the result is classified as inconclusive when assay performance or uncertainty prevents either conclusion. Step 5 matches the demonstrated evidence to the intended claim. Model qualification, external association/prospective predictive validity, and clinical utility are context-specific, non-cumulative evidence domains; prospective predictive validity requires a locked assay/analysis rule evaluated without retuning in an independent validation cohort. The workflow ends in a supported, bounded claim, with unsupported extrapolations stated explicitly. Abbreviations: ECM, extracellular matrix; MPS, microphysiological system.

**Table 1:** Decision-oriented biological requirements for physiologically relevant tumour models. Pre-analytical specimen state is a cross-cutting input; each domain is paired with the minimum qualification evidence and the principal inference to avoid.

Biological Requirement	Required Biology	Minimum Qualification Evidence	Do Not Infer
Pre-analytical specimen state (not the 7th state) [36,37,39,40]	Decision-relevant specimen state retained or deliberately reconstructed	Site/region; tumour content; prior therapy Ischaemia, transport, and processing Passage/cryopreservation; time to assay Parental/time-zero check when relevant	Fresh or patient-derived material reproduces the parental state.
Multicellular organization and heterogeneity [30,31,34,35,39]	Relevant malignant and non-malignant states Biologically meaningful spatial relationships	Cell/state identity Spatial organization Interaction-dependent perturbation	Multiple cell types alone demonstrate functional multicellularity
ECM architecture and mechanics [26,27,44,45,46]	Question-relevant composition, ligand presentation, mechanics/degradability, remodelling and transport	Mechanical characterization Remodelling Mechanotransduction or transport response	A named hydrogel or 3D scaffold alone demonstrates ECM fidelity
Stromal regulation [28,51,52,53,54]	Functional tumour–stroma communication affecting phenotype, matrix, metabolism or treatment response	Defined stromal origin/state Compartment-specific perturbation Effect on tumour, matrix or drug response	Adding fibroblasts alone demonstrates stromal relevance
Vascular and interstitial transport [56,57,58,59,60]	Perfusable barrier, flow, permeability and tissue delivery when transport is decision-critical	Lumen continuity and tracer perfusion Barrier/permeability measurement Delivered-dose characterization	Endothelial markers or lumen-like morphology alone demonstrate vascular function
Immune regulation and communication [61,62,63,64,65]	Question-relevant recognition, recruitment, infiltration, suppression or killing	Migration/contact Antigen-specific activity Cytokine or suppressive function	Immune-cell presence or bulk cytokine release alone demonstrates immune function
Metabolic regulation and biochemical gradients [66,67,68,69,70]	Spatial oxygen, nutrient, pH or metabolite conditions linked to cellular adaptation	Direct gradient/metabolite mapping Regional cell-state or treatment-response analysis	Construct size or bulk metabolite measurement alone demonstrates a spatial gradient

**Abbreviations and notation:** 3D, three-dimensional; ECM, extracellular matrix; pH, acidity or alkalinity measure.

**Table 2:** Decision-oriented comparison of major multicellular tumour-model formats. Biological configuration and implementation strategy are orthogonal descriptors; see Figure 3.

Format	Core Capability	Best Use	Main Limitation
Multicellular spheroid	Controlled 3D aggregation; diffusion-dependent physiology; scalable co-culture	Scalable 3D pharmacology; rapid matched tumour–stroma comparisons	Native ECM, perfusion and recruitment are not inherent
Tumour organoid	Renewable patient-derived tumour material; repeated experiments; configurable co-culture	Disease modelling; biobanking; repeated patient-derived pharmacology	Establishment selection and non-epithelial retention are configuration-specific
Explant/tissue slice	Short-term native architecture and resident multicellular context	Spatial pharmacodynamics; acute immune or tissue-response studies	Finite tissue, injury, drift and absent systemic input limit duration and scale
Matrix-engineered/bioprinted	Controlled matrix, mechanics, geometry and cell placement; dECM may retain source-matrix cues; light-based fabrication where relevant	Mechanobiology; invasion; spatial reconstruction; local exposure studies	Matrix source/fabrication and compound transport can alter phenotype and apparent potency
Tumour-on-chip/cancer MPS	Defined compartments, flow, barriers, gradients and dynamic transport	Delivery, barrier, trafficking, gradient and exposure questions	Hydraulics, device pharmacokinetics and biological loading require configuration-specific qualification

**Table 4:** Application-level decision requirements, priority readouts, and principal interpretive boundaries for physiologically relevant tumour models.

Application	Decisive Function	Priority Readouts	Interpretive Boundary
Candidate prioritization	Tissue-state-dependent efficacy, selectivity and delivered exposure	Dose–response/apoptosis Longitudinal control/regrowth Free/delivered exposure; penetration; compound/carrier recovery	Qualify exposure, delivery and selectivity; does not reproduce organism-level PK or whole-body toxicity
Resistance mechanisms	Adaptive tumour and microenvironmental response over time	State transition Mechanistic perturbation/stromal rescue Regrowth; clonal or phenotypic change	Mechanistic attribution requires perturbation and recovery; resistance capture may be drug-specific
Immuno-oncology	Recognition, recruitment, infiltration, suppression, persistence and killing	Migration/endothelial crossing Contact/cytotoxicity Persistence; cytokines; antigen escape	Systemic priming, lymphoid-organ interactions, PK and immune toxicity remain incomplete
Functional precision oncology	Patient-specific response within a clinically relevant decision window	Assay success/full denominator Ranked response/repeatability/turnaround Treatment-specific outcome association	Predictive validity requires a locked assay/rule in an independent validation cohort; association is not clinical utility

**Table 5:** Concise model-class quality-control priorities. Expanded reporting variables, controls, and failure modes are provided in Supplementary Table S11.

Model Class	Minimum QC/Reporting	Key Failure Mode
All patient-derived models	Site/region; tumour content; prior therapy Collection/transport/processing; passage/cryopreservation Assay denominator; identity; viable tumour fraction; time to result	Sampling/ischaemia bias Selective loss/overgrowth or culture drift Excluding failed/late specimens
Multicellular spheroids	Cell source; aggregation/seeding; size Viability/growth; internal state; plate variation Exposure/washout; assay dynamic range	Aggregation/gradient variability Segmentation or metabolic-readout artefact
Tumour organoids	Tissue source; establishment denominator Matrix/medium; passage; identity/stability Decision-window completion	Culture selection/drift Stromal loss or normal-cell overgrowth Establishment failure/delay
Explants/tissue slices	Region; thickness/size; processing interval Tumour content; viability/injury; assay-window stability Exposure route/penetration	Ex vivo injury or diffusion artefact Regional sampling bias Rapid compartment loss
Matrix-engineered/bioprinted	Material/tissue source; dECM processing where used Mechanics/geometry/fabrication; photocure variables where relevant Porosity/diffusion; delivered exposure; compound recovery	Batch/source or residual-processing artefact Binding/fabrication stress Diffusion-driven potency shift
Tumour-on-chip/vascularized	Device geometry/material; flow/pressure Lumen/perfusion/barrier/permeability Dosing route; delivered exposure; run stability	Adsorption/bubbles/unintended flow Hydraulic variability/leakage Non-functional endothelium
Immune-integrated	Immune source/HLA; antigen/activation; ratios Migration/engagement/persistence; specificity Medium/cytokines; timing/route	Alloreactivity or overactivation/exhaustion Medium/cytokine artefact Restricted migration/loss of function

Abbreviation: HLA, human leukocyte antigen; dECM, decellularized extracellular matrix.

**Table 6:** Operational definitions for the non-cumulative evidence domains shown in Figure 4. Each domain is considered separately; the table states the minimum evidence, the supported claim, and the principal extrapolation that does not follow.

Evidence Domain	Minimum Evidence	Supports	Does Not Support
Analytical/operational qualification	Repeatability/precision; control separation Pre-analytical robustness; failures/missingness Delivered exposure; endpoint stability; transfer/version control	Assay performance and implementation feasibility	Biological relevance, external prediction, or clinical utility
Track A: Identity/structural fidelity	Identity/composition evidence Architecture or spatial correspondence Parental/time-zero comparison where relevant	Structural correspondence in the tested model/time window	Biological function or altered pharmacological response
Track A: Functional fidelity	Direct measurement of the claimed function Mechanism-specific perturbation for causal claims	Specified biological function	External prediction
Track A: Comparative pharmacological response	Matched configuration or justified comparator Defined/delivered exposure Reproducible prespecified response difference	Configuration-dependent pharmacological response	In vivo or clinical association
Track B: External association	Independent claim-matched benchmark Full denominators/failures where relevant	Association with the external benchmark	Prospective predictive validity or clinical utility
Track B: Prospective predictive validity	Locked assay, analysis, endpoint, and prediction rule Independent treatment-specific validation cohort No retuning	Prediction for the locked rule in the validated context	Clinical utility
Track B: Clinical utility	Prespecified assay-guided decision rule Documented management impact and comparator strategy Clinically relevant decision/outcome benefit	Clinical utility in the validated context	Universal transfer beyond the validated context

## Data Availability

No new data were created or analysed in this study. Data sharing is not applicable to this article.

## Supplementary Materials

The following supporting information can be downloaded at https://www.mdpi.com/article/10.3390/pharmaceutics18101227/s1, Supplementary Methods S1: Structured critical narrative review: search, evidence selection, and translational appraisal strategy; Table S1: Information sources, coverage, and use; Table S2: Representative Boolean concept blocks used in the iterative search; Table S3: Evidence-selection, conflict handling, citation verification, and commercial-platform eligibility; Table S4A: Design and implementation characteristics of representative functional precision-oncology studies. Reference numbers correspond to the final main-manuscript bibliography; Table S4B: Threshold status, operational evidence classification and principal interpretive boundary for the studies summarized in Table S4A. Evidence labels follow the non-cumulative codebook in Table S8; Table S5A: Provider-reported commercial architecture and intended use. Official provider webpages are linked directly and are treated as time-stamped descriptive sources rather than numbered scientific evidence; Table S5B: Platform-specific oncology implementation evidence, provenance and author-derived evidentiary boundary. A publication documents the tested implementation only; peer-reviewed use is not equated with independent validation of every configuration; Table S6: Study-level evidence classification of representative models discussed in the main text. Reference numbers in this table correspond to the final main-manuscript bibliography (197 references); Table S7: Revision-stage reproducible verification log for the provider-specific commercial evidence map (initial verification: 4 September 2026; targeted PhysioMimix^®^ Core update: 15 September 2026; Round 2 PubMed/MEDLINE recheck: 18 September 2026). Final commercial-platform and bibliographic audit: 22 September 2026; Table S8: Operational evidence-classification codebook for the non-cumulative two-track framework; Table S9: Reusable biology-to-decision record for mechanism-guided and discovery-oriented model selection; Table S10: Revision-stage targeted scientific verification for reviewer-requested content expansion (search date: 4 September 2026); Table S11: Expanded model-class reporting and quality-control checklist. This table provides the detailed variables, critical quality attributes, controls, and frequent failure modes summarized in main-text Table 5. The items are fit-for-purpose recommendations rather than universal acceptance thresholds.

## Abbreviations

The following abbreviations are used in this manuscript:

3D	Three-dimensional
dECM	Decellularized extracellular matrix
DLP	Digital light processing
AI	Artificial intelligence
APC	Adenomatous polyposis coli
ATP	Adenosine triphosphate
CAR-T	Chimeric antigen receptor T cell
ECM	Extracellular matrix
FDA	U.S. Food and Drug Administration
FPO	Functional precision oncology
HLA	Human leukocyte antigen
MEDLINE	Medical Literature Analysis and Retrieval System Online
MPS	Microphysiological system
NAMs	New Approach Methodologies
NK	Natural killer
OECD	Organisation for Economic Co-operation and Development
PD-1	Programmed cell death protein 1
pH	Measure of acidity or alkalinity
PRISMA	Preferred Reporting Items for Systematic Reviews and Meta-Analyses
SLA	Stereolithography
TAZ	Transcriptional coactivator with PDZ-binding motif
VEGFR2	Vascular endothelial growth factor receptor 2
YAP	Yes-associated protein
