# Supplementary material for: A Biology-Driven Framework for Selecting, Engineering, and Validating Physiologically Relevant Multicellular Tumour Models
Source: Pharmaceutics. 2026 Sep 27;18(10):1227. doi: 10.3390/pharmaceutics18101227 (PMC13708530; doi:10.3390/pharmaceutics18101227)
Supplement: Supplementary file 1 [file pharmaceutics-18-01227-s001.zip › pharmaceutics-4532818-supplementary.pdf]

Supplementary Materials

A Biology-Driven Framework for Selecting, Engineering, and Validating Physiologically Relevant Multicellular Tumour Models

Ying-Ta Wu[1,†], Darshan T. Govindaraju[1,†], Girija M. Lakshmikanthacharya[2], Tzu-Yu Hsu[1], Chen-Fang Lin[1], Shashwat Sharma[3] and Anilkumar T. Shivanna[1,*]

[1] MatchCure Inc., 3F., No. 632, Guangfu South Road, Da'an District, Taipei City 10695, Taiwan

[2] Independent Researcher

[3] International Ph.D. Program in Medicine, College of Medicine, Taipei Medical University, Taipei 110, Taiwan

† These authors contributed equally to this work.

*** Correspondence:** anilkumar.shivanna@imatchcure.com

Supplementary Methods S1. Structured critical narrative review: search, evidence selection, and translational appraisal strategy

This supplement documents the purposive, iterative search and appraisal procedures used in the structured critical narrative review. The tables provide the information sources, representative Boolean concept blocks, evidence-selection rules, the separate procedure used to identify commercial implementations, a structured appraisal of representative functional precision-oncology studies, a source-separated provider-specific commercial evidence map, study-level evidence classifications for representative models discussed in the main text, a reproducible provider-verification log, an operational evidence-classification codebook, a reusable biology-to-decision record for mechanism-guided or discovery-oriented model selection, and the exact targeted revision-stage searches used to expand decellularized/patient-derived extracellular matrix, light-mediated bioprinting, and pharmaceutical exposure/drug-delivery coverage. They are intended to make the scope, evidence-appraisal process, provider-reported description, peer-reviewed evidence provenance, author-derived interpretation, commercial-selection-bias safeguards, translational evidence boundaries, and proposed decision logic transparent; they do not imply protocol registration, exhaustive retrieval, formal risk-of-bias assessment, quantitative evidence synthesis, product endorsement, head-to-head platform ranking, or prospective validation of the proposed framework. Commercial provider webpages are linked directly in Table S5 and are not included in the numbered scientific bibliography. The generative-AI disclosure in Section 1.4 and the Acknowledgments of the main manuscript also applies to these Supplementary Materials.

Reproducibility statement. The original narrative search was iterative and purposive and was not prospectively logged query-by-query; complete historical query strings and screening counts are therefore unavailable and were not retrospectively reconstructed. To make the literature assembly transparent, Table S1 reports information sources and date coverage, Table S2 reports the representative Boolean concept blocks used, Table S3 reports evidence-selection and classification rules, and Tables S7 and S10 provide the exact date-stamped queries used for the targeted revision-stage commercial and scientific verification searches.

Table S1. Information sources, coverage, and use

Source category	Source or route	Coverage / final check	Purpose and boundary
Scientific literature	PubMed/MEDLINE	Database inception to 31 July 2026	Primary search source for original studies, reviews, and bibliographic metadata. No lower year limit was imposed.
Revision-stage targeted scientific verification	PubMed/MEDLINE exact topic queries for dECM/patient-derived ECM, light-mediated bioprinting, and pharmaceutical exposure/drug-delivery modelling	Rerun 4 September 2026; eligible scientific records restricted to publication/e-publication on or before 31 July 2026	Used to address peer-review requests for topic expansion while preserving the original scientific-literature cutoff. Exact queries and retained sources are recorded in Table S10.
Full-text and metadata verification	Publisher websites and DOI landing pages, used as required	Checked during synthesis through 31 July 2026; reviewer-flagged bibliographic metadata and commercial-map references rechecked 4 September 2026	Used to retrieve full text, confirm article details, and verify whether cited sources were appropriate for the associated statements.
Citation tracking	Backward and forward tracking from landmark studies, recent original articles, and major reviews	Iterative through 31 July 2026	Used to identify foundational studies, closely related platform papers, subsequent validation studies, and conflicting evidence.
Standards and regulatory material	Official FDA, EMA, OECD, and CEN-CENELEC sources	Scientific synthesis through 31 July 2026; document availability and current versions rechecked 5 August 2026	Used only for regulatory status, quality principles, standardization, and stated programme scope.
Commercial platform documentation	Official provider websites and publicly accessible product/application documents	Initial separate review on 1 August 2026; provider pages and implementation evidence rechecked 4 September 2026; targeted PhysioMimix® Core implementation-evidence update 15 September 2026	Used only for provider-reported architecture, availability, compatibility, operational format, and stated intended use; provider claims were separated from journal and conference-level implementation evidence.

Abbreviations: CEN, European Committee for Standardization; CENELEC, European Committee for Electrotechnical Standardization; DOI, digital object identifier; EMA, European Medicines Agency; FDA, U.S. Food and Drug Administration; MEDLINE, Medical Literature Analysis and Retrieval System Online; OECD, Organisation for Economic Co-operation and Development.

Table S2. Representative Boolean concept blocks used in the iterative search

Theme	Representative search structure	Evidence sought
General model landscape	(tumor model* OR tumour model* OR spheroid* OR organoid* OR explant* OR "precision-cut slice*" OR "tumor-on-chip" OR "tumour-on-chip" OR "cancer-on-chip" OR "microphysiological system*" OR bioprint*)<br>AND (physiolog* OR multicellular OR microenvironment OR "context of use")	Platform definitions, comparative capabilities, limitations, and context-of-use concepts.
Multicellular organization and heterogeneity	(tumor OR tumour OR cancer)<br>AND (heterogene* OR "spatial organization" OR "cellular neighbourhood*" OR "cell-cell interaction*" OR "single-cell" OR "spatial transcriptom*")<br>AND (model* OR organoid* OR spheroid*)	Cell states, spatial relationships, multicellular interactions, and preservation or reconstruction of heterogeneity.
Extracellular-matrix architecture and mechanics	(tumor OR tumour OR cancer)<br>AND (extracellular matrix OR ECM OR dECM OR "decellularized extracellular matrix" OR "decellularised extracellular matrix" OR "patient-derived extracellular matrix" OR stiffness OR viscoelastic* OR mechanotransduction OR crosslink* OR degradab*)<br>AND (3D model* OR organoid* OR spheroid* OR hydrogel* OR scaffold* OR bioprint*)	Matrix composition, stiffness, viscoelasticity, remodelling, mechanotransduction, tissue/patient-derived ECM preservation, invasion, transport, and treatment-response effects.
Stromal regulation	(tumor OR tumour OR cancer)<br>AND (fibroblast* OR stromal OR mesenchymal OR adipocyte* OR "stromal signalling" OR "matrix remodelling")<br>AND (3D model* OR organoid* OR spheroid* OR hydrogel* OR explant* OR chip)	Stromal cell identity and state, reciprocal signalling, matrix remodelling, metabolic support, invasion, immune regulation, and treatment response.
Vascular and interstitial transport (including lymphatic extensions)	(tumor OR tumour OR cancer)<br>AND (vascular* OR endothelial OR perfusion OR permeability OR "interstitial flow" OR lymphatic* OR transport OR "drug delivery")<br>AND (chip OR microfluidic* OR model* OR organoid* OR spheroid*)	Perfusion, barrier function, transport, heterogeneous exposure, immune trafficking, and device pharmacokinetics.
Immune regulation and communication	(tumor OR tumour OR cancer)<br>AND (immune OR T cell OR CAR-T OR NK OR macrophage OR checkpoint OR cytokine OR chemokine)<br>AND (organoid* OR spheroid* OR explant* OR chip OR co-culture)	Recognition, recruitment, infiltration, suppression, contact, killing, persistence, and immune-compatible culture conditions.
Metabolic regulation and biochemical gradients	(tumor OR tumour OR cancer)<br>AND (metabol* OR hypoxi* OR oxygen OR nutrient* OR pH OR lactate OR gradient*)<br>AND (3D model* OR spheroid* OR organoid* OR chip OR microenvironment)	Measured gradients, metabolic exchange, regional adaptation, and treatment-response consequences.
Biofabrication and light-mediated bioprinting	(tumor OR tumour OR cancer)<br>AND (bioprint* OR biofabricat*)<br>AND (extrusion OR "digital light processing" OR DLP OR stereolithograph* OR SLA OR "light-based" OR photopolymer* OR photocur*)	Extrusion and light-mediated fabrication strategies, spatial resolution, photocurable bioinks, vascular/channel geometry, fabrication-induced stress, and pharmacological testing.
Applications and translation	(patient-derived OR functional precision oncology OR functional precision medicine OR comparative oncology OR pharmaceutic* OR "drug delivery" OR nanomedicine OR pharmacokinetic*)<br>AND (spheroid* OR organoid* OR explant* OR chip OR microfluidic* OR drug screen* OR treatment response OR predictive OR clinical utility OR penetration OR transport)	Drug discovery, delivery and transport, exposure-dependent pharmacology, resistance, immuno-oncology, patient-specific testing, companion-animal studies, and outcome association.
Quality, validation, and regulation	(microphysiological system* OR organ-on-chip OR tumor model* OR tumour model*)<br>AND (validation OR reproducib* OR standard* OR quality control OR fit-for-purpose OR NAM OR GIVIMP OR qualification OR regulatory)	Analytical performance, reproducibility, acceptance criteria, reporting, transferability, and regulatory context.
Context-specific extension terms	(tumor OR tumour OR cancer)<br>AND (neural OR nerve OR adipose OR endocrine OR microbiome OR "metastatic niche" OR pharmacokinetic*)<br>AND (model* OR organoid* OR chip OR explant*)	Processes not assumed to be fully represented by the six core domains and requiring linked or additional modules.

Abbreviations and notation: 3D, three-dimensional; CAR-T, chimeric antigen receptor T-cell; dECM, decellularized extracellular matrix; DLP, digital light processing; ECM, extracellular matrix; GIVIMP, Good In Vitro Method Practices; NAM, New Approach Methodology; NK, natural killer; pH, acidity or alkalinity measure; SLA, stereolithography. AND and OR are Boolean search operators.

Table S3. Evidence-selection, conflict handling, citation verification, and commercial-platform eligibility

Process	Rule applied	Interpretive boundary
Mechanistic / functional claims	• Prioritize original studies<br>• Direct function-specific readout, matched configuration or perturbation	Markers or structural resemblance alone are not proof of function.
Platform performance / pharmacology	• Report operational variables and controls<br>• Use delivered exposure and justified comparators where relevant	A model-to-model difference supports comparative pharmacological-response evidence only for the tested configuration, exposure and endpoint.
Translational claims	• Prioritize patient-derived evidence	Outcome association is not evidence that assay use improves outcome.

Process	Rule applied	Interpretive boundary
	• Separate external association, predictive validity and clinical utility	
Reviews / consensus / standards	• Use authoritative reviews for field concepts<br>• Use consensus, standard and regulatory documents for quality/adoption statements	Do not substitute secondary sources for specific mechanistic or treatment-response evidence.
Duplicate / overlapping reports	• Use the most complete peer-reviewed report<br>• Retain conference abstracts only when no full report is available; label them explicitly	Reports from one cohort are not independent validation without independent samples/analyses.
Conflicting evidence	• Retain and contextualize disagreement by tumour, specimen, configuration, exposure, endpoint and validation design	No pooled adjudication or formal evidence grade was assigned.
Citation verification	• Check PubMed/publisher metadata<br>• Verify claim relevance against abstract/full text; place citations adjacent to the supported statement<br>• Audit duplicate DOI, undefined citations and uncited bibliography entries	Bibliographic correctness, citation relevance and mechanical consistency are separate checks.
Commercial inclusion	• Active public documentation<br>• Oncology application + engineering capability beyond monolayer<br>• Adequate architecture information	Inclusion is descriptive, not validation, regulatory qualification or endorsement.
Commercial exclusion	• No oncology-specific documentation<br>• Unavailable/discontinued or duplicate families<br>• Insufficient public technical description	The commercial set is representative, not exhaustive.
Commercial extraction	• Provider/product and architecture<br>• Provider-documented intended use<br>• Implementation evidence + provenance + official source	Provider claims, implementation evidence and author interpretation are kept separate; no ranking.
Platform-specific evidence search	• Use exact provider/product oncology queries in Table S7<br>• Cross-check publisher/DOI and official provider pages<br>• Separate journal and conference evidence; review affiliations/COI	One publication documents one implementation; failure to identify a paper is not evidence of absence.
Commercial-selection-bias safeguards	• Apply identical eligibility/extraction fields<br>• Public sources only; no provider contact/unpublished information<br>• Exclude MatchCure; no ranking	Safeguards reduce but cannot eliminate affiliation-related selection/interpretation bias.
Evidence-domain assignment and verification	• The corresponding author assigned and systematically verified all labels in Tables S4B and S6 against the operational definitions in Table S8<br>• Multiple non-cumulative labels were retained only when separately supported<br>• Borderline or ambiguous cases were handled conservatively: the more conservative classification was used unless the minimum criterion for the higher domain was clearly met	The complete classification set was rechecked for internal consistency by the corresponding author. No separate second-author or formal inter-rater verification was used.
Final consistency audit	• All assigned labels and interpretive boundaries were rechecked against Table S8 before resubmission<br>• Internal inconsistencies identified during this audit were corrected	This final audit verifies internal consistency; it is not an inter-rater reliability analysis.

Note: The original scientific-literature searches were iterative and were refined as new concepts, landmark studies, and citation networks were identified. The theme labels use the preferred manuscript terminology. Alternative spellings and field synonyms—including tumor/tumour, tumor-on-chip/tumour-on-chip/cancer-on-chip, vascularization, perfusion, microphysiological system, decellularized/decellularised ECM, and light-based bioprinting—were retained as retrieval terms. These concept blocks document the principal original search logic but are not a complete date-stamped export of every historical query, and complete screening counts were not retained. They therefore support a structured critical narrative review rather than a systematic-review claim. In contrast, the revision-stage commercial-platform verification was rerun using the exact date-stamped queries reported in Table S7, and the targeted scientific expansion for dECM/patient-derived ECM, light-mediated bioprinting, and pharmaceutical exposure/drug-delivery modelling was rerun using the exact queries reported in Table S10. Scientific records from the targeted expansion were restricted to publication or electronic publication on or before 31 July 2026. Historical query logs and screening counts that were not retained were not retrospectively reconstructed or estimated; instead, the revised manuscript explicitly limits field-level inference and reports exact revision-stage verification searches where they were actually rerun. Abbreviations: MEDLINE, Medical Literature Analysis and Retrieval System Online. AND and OR are Boolean search operators.

Table S4A. Design and implementation characteristics of representative functional precision-oncology studies. Reference numbers correspond to the final main-manuscript bibliography.

Table S4A reports study design, assay-success denominators and treatment allocation. Threshold status and evidence interpretation are separated into Table S4B so that the clinical-design record can be read independently from the authors' evidence labels.

Study	Tumour / assay	Design / cohort	Assay-success denominator	Treatment matching / allocation

Study	Tumour / assay	Design / cohort	Assay-success denominator	Treatment matching / allocation
Ooft et al. [127]	Metastatic colorectal cancer; biopsy-derived organoids and regimen-specific growth-rate assays	Prospective multicentre observational study; 61 patients, 67 biopsies; 35 cultures from 29 patients evaluable	40 cultures from 34 patients established from 63 biopsies placed in culture (approximately 63%); 35 cultures/29 patients evaluable	Patients received standard-of-care irinotecan, fluoropyrimidine–irinotecan, or fluoropyrimidine–oxaliplatin independently of assay
Shuford et al. [176]	Newly diagnosed ovarian cancer; rapid patient-derived three-dimensional spheroid assay	Prospective blinded, non-interventional prediction; 44 clinically evaluable patients; seven-day reporting window	92 eligible; 83/92 tissues (90%) successfully tested; 44 patients had sufficient clinical-response data for prediction analysis (35 adjuvant and 9 neoadjuvant)	Predictions were stored while patients received standard first-line therapy; results were not used for treatment selection
Cartry et al. [175]	Advanced colorectal cancer; expandable patient-derived-organoid chemogram against 25 approved drugs	Prospective feasibility and observational follow-up; 25 patient-derived organoids established; eight patients had matched drug and outcome data	Core-biopsy take-on rate 61.5% (8/13); 25 total patient-derived organoids established from mixed surgical and biopsy specimens	Eight patients received standard-care drugs represented in the panel; the chemogram did not orient treatment
Boilève et al. [174]	Advanced refractory pancreatic ductal adenocarcinoma; patient-derived-organoid chemogram against approved agents	Prospective observational implementation study; 87 patients sampled, 54 patient-derived organoids generated, 34 with full clinical follow-up	54/87 patient-derived-organoid take-on (62%); 34/54 had evaluable matched clinical follow-up	Some patients received a chemogram hit and others a non-hit during routine management; assignment was not protocol mandated or randomized
Lee et al. [131]	Epidermal growth factor receptor (EGFR)-mutant advanced non-small-cell lung cancer; malignant-pleural-effusion-derived lung organoids and a study-specific drug-response prediction index	Prospective observational comparison; 26 effusions collected, 14 EGFR-mutant cases included after growth, quality-control, and disease exclusions	14/26 included in the final analysis; exclusions included non-EGFR disease, insufficient organoid growth, and quality-control failure	EGFR-targeted tyrosine kinase inhibitor treatment was selected clinically according to genotype; organoid results were compared with subsequent Response Evaluation Criteria in Solid Tumours (RECIST) response
Smabers et al. [130]	Metastatic colorectal cancer; biopsy-derived patient-derived-organoid panel including the patient's treatment	Prospective multicentre observational study; 232 patient inclusions, 205 biopsies; interim treatment-response analysis of 42 patient-derived organoids	107/205 patient-derived organoids established overall (52%); establishment improved from 22% to 75% during optimization; 42 screened in the interim analysis	Biopsy preceded a new standard systemic treatment; assay result was evaluated against treatment response, not used to allocate therapy

Table S4B. Threshold status, operational evidence classification and principal interpretive boundary for the studies summarized in Table S4A. Evidence labels follow the non-cumulative codebook in Table S8.

Study	Threshold status	Evidence classification	Principal boundary
Ooft et al. [127]	Classifier and cut-offs developed within the study (for example, irinotecan growth-rate cut-off) and evaluated by internal cross-validation; not independently locked	Track A: comparative pharmacological response evidence. Track B: external association with prospective observational predictive-performance analysis; not prospective predictive validity.	Regimen-specific performance; internally developed cut-offs and no assay-guided allocation. Prospective enrolment does not establish predictive validity.
Shuford et al. [176]	Drug-specific cut-offs were derived or confirmed using available cohort data; not an independently locked external threshold	Track A: comparative pharmacological response evidence. Track B: external association with prospective blinded predictive-performance analysis; not prospective predictive validity.	Blinded prediction did not guide care; threshold development/confirmation and validation were not independent; no utility comparison.
Cartry et al. [175]	Hit score >1.9 was defined from the cohort score distribution; no independent threshold-locking cohort	Track A: comparative pharmacological response evidence. Operational descriptor: prospective feasibility/take-on and turnaround. Track B: limited prospective external association; not prospective predictive validity or clinical utility.	Median turnaround six weeks; only eight matched outcomes; treatment independent of assay; no independently locked rule or utility assessment.
Boilève et al. [174]	Outlier-based hit definition applied within the study; no independently validated clinical decision threshold	Track A: comparative pharmacological response evidence. Operational descriptor: implementation feasibility/take-on. Track B: prospective observational external association with non-randomized treatment matching; not prospective predictive validity or clinical utility.	Treatment matching was non-randomized and not protocol mandated; association is vulnerable to treatment availability/selection and does not establish predictive validity or utility.
Lee et al. [131]	Study-specific index cut-off (−0.17) identified within the dataset; no independent locked validation cohort	Track A: comparative pharmacological response evidence. Track B: prospective observational external association with predictive-performance analysis; not prospective predictive validity.	Small selected cohort; index and cut-off developed within the dataset; no independent validation cohort or assay-guided allocation.
Smabers et al. [130]	Diagnostic cut-offs and receiver-operating-characteristic performance evaluated within the cohort; no external independently locked threshold reported	Track A: comparative pharmacological response evidence. Track B: prospective multicentre external association with predictive-performance analysis; not prospective predictive validity.	Interim subset and evolving workflow; ROC/cut-offs evaluated within the cohort; no independently locked rule or utility test.

Abbreviations: EGFR, epidermal growth factor receptor; RECIST, Response Evaluation Criteria in Solid Tumours. "Clinical utility" is reserved for evidence that use of an assay result changes management and improves a clinically relevant outcome compared with a prespecified alternative strategy.

Table S5A. Provider-reported commercial architecture and intended use. Official provider webpages are linked directly and are treated as time-stamped descriptive sources rather than numbered scientific evidence.

Commercial-source layers are separated across two tables for readability and bias control. Table S5A contains only provider-reported architecture and intended use. Table S5B contains the oncology implementation identified by the defined verification search, evidence provenance and the authors' evidentiary boundary. Provider information was initially checked on 1 August 2026 and rechecked on 4 September 2026. A targeted PhysioMimix® Core implementation-evidence recheck was performed on 15 September 2026. The mapping is descriptive, representative, non-exhaustive and non-ranking; MatchCure products/services were excluded and no provider supplied unpublished information. Exact verification queries are reported in Table S7.

Product / provider and source	Provider-reported architecture	Provider-documented intended use
OrganoPlate® / MIMETAS<br>Official provider webpage	Microfluidic well plate with membrane-free gel patterning and gravity- or unidirectional perfusion; microplate-scale parallelization.	Perfused 3D tumour models, invasion, vascular interaction, immune trafficking, and screening with user-selected cells and matrices.
Organ-Chip® / Emulate<br>Official provider webpage	Two-channel tissue–vascular interface chip with controlled flow and dedicated culture instrumentation.	Tumour–vascular interfaces, dynamic exposure, intravasation, immune trafficking, and patient-derived tissue configurations.
idenTx® / AIM Biotech<br>Official provider webpage	Central hydrogel region flanked by perfusion channels; gravity-driven flow and parallel assay sites.	Invasion, angiogenesis, extravasation, chemotaxis, immune-cell migration, and primary or patient-derived cell models.
3D InSight™ / Akura™ / InSphero<br>Official provider webpage	Hanging-drop and low-attachment spheroid or microtissue plates in 96- and 384-well formats.	Parallel tumour spheroids, drug testing, patient-derived microtissues, and tumour–immune co-culture.
HUMIMIC® / TissUse<br>Official provider webpage	Recirculating multi-compartment or multi-organ chip with configurable tissue chambers.	Inter-organ exposure, metabolism, efficacy–toxicity coupling, and complex tissue interactions.
PhysioMimix® Core / CN Bio<br>Official provider webpage	Perfused multi-well MPS with recirculating medium and insert-based tissue compartments.	Perfused tumour, metastatic-niche, immune-trafficking, and efficacy–toxicity workflows.
DynamicOrgan® TME Kit / Dynamic42<br>Official provider webpage	Three-channel perfused biochip with matrix-embedded tumour spheroids and optional microvasculature.	Vascularized tumour modelling, dynamic dosing, and immune invasion.
Human-on-a-Chip® / Hesperos<br>Official provider webpage	Reconfigurable pumpless multi-organ MPS connecting tumour and normal-tissue modules.	Concurrent antitumour efficacy, off-target toxicity, and therapeutic-index assessment.
MIVO® / React4life<br>Official provider webpage	Millifluidic chamber supporting 3D tissues under dynamic flow with optional immune-cell circulation.	Dynamic tumour testing, tissue-scale exposure, biopsies or organoids, and immune infiltration.
CurioChip® / Qureator<br>Official provider webpage	Open-channel MPS integrating patient-derived tumour, stromal, endothelial, and immune components with computational analysis.	Metastasis, vascularized tumour–immune interaction, combination testing, and biomarker exploration.

Table S5B. Platform-specific oncology implementation evidence, provenance and author-derived evidentiary boundary. A publication documents the tested implementation only; peer-reviewed use is not equated with independent validation of every configuration.

Product / provider	Oncology implementation identified	Evidence provenance	Author-derived boundary
OrganoPlate® / MIMETAS	Lanz et al. [150], perfused breast-cancer therapy-response model.	Provider-associated development.	One breast-cancer configuration; does not independently validate all oncology uses.
Organ-Chip® / Emulate	Hassell et al. [146] and Pal et al. [151], lung-cancer and patient-derived oesophageal-adenocarcinoma chips.	Provider-associated development context disclosed.	Evidence is disease- and configuration-specific and does not establish a standardized predictive assay.
idenTx® / AIM Biotech	Miller et al. [152], renal-cell-carcinoma spheroids and tumour-reactive T cells in idenTx 3.	External academic use.	Commercial availability does not validate delivered exposure, vascular function, or predictive performance.
3D InSight™ / Akura™ / InSphero	Hofmann et al. [153], patient-derived breast-tumour spheroids using InSphero and comparator plate formats.	External academic use.	The study supports feasibility of a spheroid workflow rather than exclusive or head-to-head validation of the commercial format.
HUMIMIC® / TissUse	Somova et al. [154], linked healthy renal epithelium and renal-cell-carcinoma model.	External academic use.	One mechanistic renal-cancer configuration does not establish organism-level physiology or general prediction.
PhysioMimix® Core / CN Bio	Bouges et al. [159], rectal patient-derived tumour-organoid-derived monolayers perfused in a PhysioMimix Barrier plate using the PhysioMimix® Core System for fractionated radiotherapy modelling. A separate 2026 PhysioMimix Core melanoma CAR-T implementation remains conference-level evidence.	External academic peer-reviewed journal protocol; no CN Bio author affiliation was reported. Separate current-Core melanoma CAR-T evidence remains conference-level;	Direct peer-reviewed use of the current Core configuration is supported for one rectal tumour-organoid-derived monolayer/barrier radiotherapy workflow. This does not validate other tumour types, 3D multicellular configurations,

Product / provider	Oncology implementation identified	Evidence provenance	Author-derived boundary
		legacy CN Bio/LiverChip literature is kept distinct.	predictive performance, or all PhysioMimix Core applications; legacy-system evidence should not be transferred automatically.
DynamicOrgan® TME Kit / Dynamic42	Deipenbrock et al. [157], multicellular PDAC tumour-microenvironment model using the Dynamic42 BC003 three-channel biochip.	Provider-associated development (Dynamic42 affiliations reported).	One PDAC fit-for-purpose BC003 configuration supports feasibility and mechanistic use but does not establish interlaboratory performance or validate all DynamicOrgan® tumour applications.
Human-on-a-Chip® / Hesperos	McAleer et al. [156], reconfigurable multi-organ system for simultaneous anticancer efficacy and off-target toxicity assessment.	Provider-associated development (multiple Hesperos affiliations reported).	The study supports one multi-organ anticancer efficacy/toxicity configuration; it does not independently validate every Human-on-a-Chip® configuration or establish prospective clinical prediction.
MIVO® / React4life	Marrella et al. [155], dynamic ovarian-cancer drug-efficacy model.	Provider-associated development.	Comparison with static culture and xenograft is specific to the tested SKOV-3/cisplatin configuration.
CurioChip® / Qureator	Kim et al. [158], AACR 2023 Curiochips PDAC tumour–stroma microphysiological-system conference abstract; no full journal oncology article was identified in the defined verification search.	Provider-associated conference-level development (Qureator affiliations reported).	Conference-level evidence supports feasibility of the reported configuration but is not equivalent to a full peer-reviewed journal validation or independent predictive-performance study.

Table S6. Study-level evidence classification of representative models discussed in the main text. Reference numbers in this table correspond to the final main-manuscript bibliography (197 references).

Evidence labels follow the non-cumulative two-track framework in Section 1.4 and the operational codebook in Table S8. Track A separates identity/structural fidelity, functional fidelity, comparative pharmacological response evidence, and cross-cutting analytical/operational qualification rather than combining structural and functional evidence. Track B uses external association, prospective predictive validity, and clinical utility. "Predictive-performance" is used only as a descriptive modifier of external association when outcome discrimination or prediction was evaluated; it does not denote prospective predictive validity unless a locked assay and rule were tested without retuning in an independent validation cohort. "None" means that the cited study did not test an independent external or clinical outcome for the claim described in the review; it does not imply poor study quality. Article-title wording was not used as an evidence label. Detailed denominators and threshold status for functional precision-oncology cohorts are provided in Tables S4A and S4B.

Study	Configuration or principal comparison	Track A — model qualification / mechanistic evidence	Track B — external / decision-performance evidence	Interpretive boundary
**Cornelison et al. [96]**	Glioblastoma-margin model with glial cells and interstitial flow; xenograft-survival benchmark	Functional fidelity; endpoint alignment	External association to a preclinical benchmark	Not patient-level prospective prediction or utility
**Haque et al. [97]**	Pancreatic organoid–stellate-cell–macrophage chip; tumour-only versus multicellular treatment response	Functional fidelity; comparative pharmacological response evidence	None	Context-specific multicellular effect; no clinical outcome benchmark
**Pyne et al. [98]**	Spheroid-on-chip with factorial interstitial-flow and hypoxia conditions	Functional fidelity	None	Mechanistic transcriptomic comparison only
**Hu et al. [99]**	Perfused tumour-slice-on-chip with preserved tissue organization	Identity/structural fidelity; functional fidelity of transport and maintenance	None	Preservation and longitudinal function do not establish prediction
**Fritschen et al. [100]**	Automated high-scale bioprinting of multicellular vascularized liver-carcinoma constructs	Analytical/operational qualification	None	Scale and manufacture are not biological or predictive validation
**Prince et al. [104]**	Engineered EKGel versus conventional patient-derived breast-organoid culture	Identity/structural fidelity; analytical/operational qualification	None	Retention of ex vivo drug-response features is not patient-outcome prediction
**Song et al. [105]**	Cervical extracellular-matrix hydrogel versus generic matrix conditions	Functional fidelity	None	Matrix-dependent phenotype only
**Singh et al. [106]**	Tunable synthetic matrix with modulus and fibroblast perturbations	Functional fidelity	None	Mechanistic matrix–stroma comparison
**Kondapaneni et al. [107]**	Hyaluronic-acid brain-metastasis model versus suspension culture and therapy response	Functional fidelity; comparative pharmacological response evidence	None	Dormancy-associated response in one engineered context
**Mondal et al. [108]**	Printed non-small-cell lung-cancer and fibroblast co-culture with bioink optimization	Analytical/operational qualification; functional fidelity	None	Printability and co-culture response do not establish external validity

Study	Configuration or principal comparison	Track A — model qualification / mechanistic evidence	Track B — external / decision-performance evidence	Interpretive boundary
**Bertillot et al. [109]**	Confined carcinoma model producing fibroblast encasement under compressive stress	Functional fidelity	None	Mechanically emergent organization in the tested construct
**Sharpe et al. [110]**	Oesophageal tumour-organoid and fibroblast assembloid	Functional fidelity	None	Transcriptional and phenotypic effects without external outcome testing
**Zhang et al. [111]**	Patient-specific lung-cancer assembloid with controlled microgels and combination testing	Identity/structural fidelity; functional fidelity; comparative pharmacological response evidence	External association (limited clinical-outcome comparison)	Seven-patient clinical-outcome comparison; small internally assessed cohort does not establish independently locked prospective predictive validity
**Moghimi et al. [112]**	Bioprinted breast-tumour-on-chip with controlled heterogeneity and chemical gradients	Functional fidelity	None	Spatially controlled response without external validation
**Nashimoto et al. [59]**	Perfusable microvascular network connected to a tumour spheroid; perfused versus non-perfused conditions	Functional fidelity; comparative pharmacological response evidence	None	Transport and delivery in one model configuration
**Pradhan et al. [60]**	Defined well- versus poorly perfused vascular geometries and anticancer-drug exposure	Functional fidelity; comparative pharmacological response evidence	None	Exposure-response comparison is not patient prediction
**Hachey et al. [113]**	Vascularized colorectal microtumour compared with 2D, non-vascularized, and in vivo responses	Identity/structural fidelity; comparative pharmacological response evidence	External association to a preclinical in vivo benchmark	“Recapitulates in vivo responses” does not mean prospective patient prediction
**Ibrahim et al. [114]**	Vascularized omentum-on-chip during ovarian metastatic colonization	Functional fidelity of barrier alteration	None	Barrier function only; no treatment-prediction claim
**Zhou et al. [64]**	Cholangiocarcinoma organoid–T-cell co-culture with medium optimization	Functional fidelity	None	Restored immune function in the configured co-culture
**Lahusen et al. [85]**	Pancreatic organoid-in-matrix platform comparing T-cell killing across extracellular environments	Functional fidelity; comparative pharmacological response evidence	None	Matrix-dependent killing does not establish clinical response
**Dekkers et al. [86]**	BEHAV3D live imaging of engineered T-cell behaviours in patient organoids	Functional fidelity	None	Behavioural-state resolution is mechanistic evidence
**Bonnet et al. [87]**	Colorectal tumour-on-chip with APC perturbation and stage-resolved T-cell engagement	Functional fidelity	None	Causal engagement mechanism without external prediction
**Maulana et al. [65]**	Perfused breast-cancer-on-chip for CAR-T trafficking, killing, and endothelial injury	Functional fidelity; comparative pharmacological response evidence	None	“Patient-specific efficacy and safety” is model-based; no patient-outcome validation
**Veith et al. [88]**	Autologous lung tumour-on-chip with anti-PD-1 and fibroblast-dependent resistance	Functional fidelity; comparative pharmacological response evidence	None in the reported cohort comparison used here	Personalized ex vivo response requires future clinical validation
**Siciliano et al. [93]**	Sensor-integrated pancreatic tumour–stroma model with longitudinal pH and apoptosis readouts	Functional fidelity; analytical/operational qualification	None	Sensor performance and mechanistic association are not prediction
**Flörkemeier et al. [118]**	Ovarian tumour spheroids with or without fibroblasts and cisplatin	Functional fidelity; comparative pharmacological response evidence	None	Heterotypic response in one assay configuration
**Chang et al. [119]**	Metastatic-brain-tumour spheroid drug response compared with clinical outcomes	Comparative pharmacological response evidence	External association (preliminary clinical-outcome comparison)	Small cohort and exclusions preclude prospective predictive validity
**Seino et al. [122]**	Pancreatic tumour organoids with niche-factor-dependence analysis	Functional fidelity of niche dependence	None	Disease-state biology, not treatment prediction
**Sachs et al. [123]**	Breast-cancer organoid biobank compared with parental tumours	Identity/structural fidelity	None	Biobank fidelity does not establish clinical response prediction
**Lee et al. [124]**	Bladder-cancer organoids with longitudinal evolution and drug-response analysis	Identity/structural fidelity; comparative pharmacological response evidence	None	Model evolution and ex vivo response without independent outcome benchmark
**Yao et al. [128]**	Locally advanced rectal-cancer organoids exposed to chemoradiation; organoid response compared with patient tumour-response benchmarks	Identity/structural fidelity; comparative pharmacological response evidence	External association with treatment-specific predictive-performance analysis	Treatment-specific association/performance only; no independently locked validation rule and no assay-guided utility test
**Ooft et al. [127]**	Prospective colorectal organoid assay compared with regimen-specific clinical response	Comparative pharmacological response evidence	External association with prospective observational predictive-performance analysis	Cut-offs developed internally; prospective observational performance does not meet the independently locked predictive-validity criterion

Study	Configuration or principal comparison	Track A — model qualification / mechanistic evidence	Track B — external / decision-performance evidence	Interpretive boundary
**Wang et al. [129]**	Lung-cancer organoid drug testing compared with treatment response in a patient cohort	Identity/structural fidelity; comparative pharmacological response evidence	External association with cohort-level predictive-performance analysis	Outcome association/performance in the reported cohort; no independently locked prospective validation rule established here
**Smabers et al. [130]**	Prospective multicentre colorectal organoid study with treatment-response analysis	Comparative pharmacological response evidence	External association with prospective observational predictive-performance analysis	Interim subset, evolving workflow, and internally evaluated cut-offs; no prospective predictive validity or clinical utility
**Steinberg et al. [177]**	Three-dimensional printed tumour-on-chip with drug screening and clinical-outcome correlation	Functional fidelity; comparative pharmacological response evidence	External association (limited clinical-outcome correlation)	Correlation does not establish prospective prediction or utility
**Shuford et al. [176]**	Prospective blinded ovarian spheroid predictions stored during standard treatment	Comparative pharmacological response evidence	External association with prospective blinded predictive-performance analysis	Blinded prospective performance, but threshold development/validation were not independent; no assay-guided allocation or utility
**Cartry et al. [175]**	Prospective colorectal organoid chemogram with limited matched clinical follow-up	Comparative pharmacological response evidence	External association (limited prospective observational follow-up)	Small matched subset; cohort-derived hit score; no independently locked rule or assay-guided allocation
**Boilève et al. [174]**	Prospective pancreatic organoid chemogram; routine receipt of hit versus non-hit treatment	Comparative pharmacological response evidence	External association (prospective observational, non-randomized treatment matching)	Non-randomized treatment matching and within-study hit definition; external association only, not predictive validity or clinical utility
**Lee et al. [131]**	EGFR-mutant lung organoids with study-specific response index and subsequent RECIST outcome	Comparative pharmacological response evidence	External association with prospective observational predictive-performance analysis	Small selected cohort with internally derived response index/cut-off; no independent locked validation cohort or utility test

Table S7. Revision-stage reproducible verification log for the provider-specific commercial evidence map (initial verification: 4 September 2026; targeted PhysioMimix® Core update: 15 September 2026; Round 2 PubMed/MEDLINE recheck: 18 September 2026). Final commercial-platform and bibliographic audit: 22 September 2026.

To make the search source explicit and reproducible, all product/provider-specific oncology queries in this table were re-run in PubMed/MEDLINE on 18 September 2026 for peer-reviewed journal literature. Because the same bibliographic database was used for every row, it is stated here rather than repeated as a separate table column. Candidate journal records were checked against publisher/DOI metadata. Official provider publication/application pages and public conference sources were checked where applicable for provider-reported or non-journal implementation evidence. The PhysioMimix® Core query had also been rerun on 15 September 2026 after targeted re-verification. The disposition below records the evidence used in Tables S5A and S5B; it is not a market-wide or exhaustive commercial-system inventory.

Product / provider	Exact verification query	Evidence disposition used in Table S5B
**OrganoPlate® / MIMETAS**	("OrganoPlate" OR MIMETAS) AND (cancer OR tumour OR tumor OR oncology)	Full journal implementation retained: Lanz et al. [150]; provider-associated development.
**Organ-Chip® / Emulate**	("Organ-Chip" OR Emulate) AND (cancer OR tumour OR tumor OR oncology)	Full journal implementations retained: Hassell et al. [146] and Pal et al. [151]; provider-associated development context.
**idenTx® / AIM Biotech**	(idenTx OR "AIM Biotech") AND (cancer OR tumour OR tumor OR oncology)	Full journal implementation retained: Miller et al. [152]; external academic use.
**3D InSight™ / Akura™ / InSphero**	("3D InSight" OR Akura OR InSphero) AND (cancer OR tumour OR tumor OR oncology)	Full journal implementation retained: Hofmann et al. [153]; external academic use.
**HUMIMIC® / TissUse**	(HUMIMIC OR TissUse) AND (cancer OR tumour OR tumor OR oncology)	Full journal implementation retained: Somova et al. [154]; external academic use.
**PhysioMimix® Core / CN Bio**	("PhysioMimix Core" OR PhysioMimix OR "CN Bio") AND (cancer OR tumour OR tumor OR oncology)	Peer-reviewed journal protocol retained: Bouges et al. [159], rectal tumour-organoid-derived monolayer/barrier model using the PhysioMimix® Core System; external academic use. Related legacy-system literature and the 2026 Core melanoma CAR-T poster were kept separate.
**DynamicOrgan® / Dynamic42**	(DynamicOrgan OR Dynamic42 OR BC003) AND (cancer OR tumour OR tumor OR oncology)	Previously omitted full journal implementation added: Deipenbrock et al. [157]; provider-associated development.
**Human-on-a-Chip® / Hesperos**	("Human-on-a-Chip" OR Hesperos) AND (cancer OR tumour OR tumor OR oncology)	Reviewer-identified full journal implementation confirmed and added: McAleer et al. [156]; provider-associated development.
**MIVO® / React4life**	(MIVO OR React4life OR React4Life) AND (cancer OR tumour OR tumor OR oncology)	Full journal implementation retained: Marrella et al. [155]; provider-associated development.

Product / provider	Exact verification query	Evidence disposition used in Table S5B
**CurioChip® / Qureator**	(CurioChip OR CurioChips OR Qureator) AND (cancer OR tumour OR tumor OR oncology)	Conference-level oncology implementation added: Kim et al. [158]; no full journal oncology article identified in the defined verification search.

Table S8. Operational evidence-classification codebook for the non-cumulative two-track framework.
Each evidence label is considered separately and is assigned only when the minimum evidence required for that domain can be verified from the source study. Labels are non-cumulative and are not ordinal grades; assignment of one label does not imply completion of another domain. When evidence is absent, incompletely reported, or ambiguous, the more conservative label is used. Predictive-performance terminology is a descriptor of outcome-association analysis, not a substitute for prospective predictive validity.

Evidence label	Minimum evidence required	Do not infer	Coding note
Analytical / operational qualification	• Repeatability/intermediate precision and control separation<br>• Pre-analytical robustness and assay failure/denominator<br>• Endpoint stability; delivered exposure/recovery; workflow/version control	Operational robustness alone does not establish biological relevance or external validity.	Assign this domain only when directly tested.
Identity / structural fidelity	• Direct identity/composition evidence<br>• Molecular/pathological or spatial/architectural correspondence<br>• Parental/time-zero comparison where relevant	Markers, morphology or patient derivation alone do not establish function.	Track A
Functional fidelity	• Direct measurement of the claimed biological function<br>• Mechanism-specific perturbation/equivalent control for causal claims	Identity, spatial organization or structural resemblance alone are insufficient.	Track A
Comparative pharmacological response	• Defined treatment exposure<br>• Justified matched comparator/configurations<br>• Clearly defined endpoint and reproducible response difference with replication/uncertainty sufficient for the claim	A treatment effect in one model alone does not establish configuration-dependent pharmacology.	Track A; not external prediction
External association	• Comparison with an independent in vivo, clinical, cross-species or other claim-matched benchmark	Association/ROC/prospective enrolment/blinding alone do not establish predictive validity.	Track B; add predictive-performance descriptor only when explicitly evaluated
Predictive-performance descriptor	• Outcome discrimination/prediction evaluated against treatment-specific outcomes<br>• Rule may be developed/tuned/confirmed within the evaluated cohort	Not a validation level; do not label predictive validity unless the independent-lock criterion is met.	Modifier of external association
Prospective predictive validity	• Assay/analysis/endpoint/prediction rule locked before evaluation<br>• Independent treatment-specific validation cohort; no retuning<br>• Blinded outcome assessment where feasible; uncertainty reported	Prospective or chronological validation is insufficient if the rule is tuned using that dataset.	Track B; context-specific to disease, treatment, population and locked rule
Clinical utility	• Prespecified assay-guided decision rule used in management<br>• Result delivery/adherence and comparator strategy documented<br>• Clinically relevant decision/outcome benefit evaluated	Feasibility, turnaround, clinician-selected matching or outcome association alone are not utility.	Track B; not mandatory for every model
None / not demonstrated	No qualifying evidence was tested or reporting was insufficient for the claim under review.	Does not imply poor study quality; it only limits the claim that can be assigned in this review.	Use conservatively; do not infer missing evidence.

Table S9. Reusable biology-to-decision record for mechanism-guided and discovery-oriented model selection.

Record field	Required entry	Decision rule / question
**Context of use and decision**	Decision to be supported, endpoint, and decision window	What scientific, pharmacological, or translational decision must the model support?
**Mechanism status**	Established / suspected / unknown	Use the mechanism-guided route only when a candidate causal mechanism can reasonably be prespecified.
**Candidate functions**	Essential / context-modifying / non-essential / uncertain	Which biological functions could alter the inference, ranking, exposure, or interpretation?
**Model format**	Spheroid / organoid / explant-slice / matrix-engineered/bioprinted / tumour-on-chip/MPS	Which physical implementation provides the required control, specimen compatibility, and analytical access?
**Biological configuration**	Tumour-only baseline / stromal / vascular / immune / metabolic/gradient-controlled / integrated multicellular	Which compartments or functions must be present within the chosen format?
**Implementation strategy**	Preservation / reconstruction / hybrid	Is the relevant biology retained from tissue, rebuilt after processing, or combined?
**Qualification evidence**	Identity/structural / functional / comparative pharmacological response / analytical/operational	What direct measurements demonstrate that the required function and assay performance are present?

Record field	Required entry	Decision rule / question
**Matched complexity test**	Module addition/removal, nested comparison, or factorial perturbation	What comparison tests whether added complexity supplies decision-relevant information?
**Retention / stop rule**	Prespecified criteria for retain / simplify / inconclusive	Retain when the added module reproducibly changes mechanism, response ranking, uncertainty, or decision-relevant interpretation. Simplify only when a qualified comparison has adequate precision and sensitivity to exclude a prespecified decision-relevant effect. Record the result as inconclusive when the experiment cannot distinguish absence of a meaningful effect from inadequate precision, sensitivity, exposure control, or assay performance.
**Claim boundary**	Supported claim plus explicit unsupported extrapolations	What does the evidence support, and what does not follow from the tested configuration?

Use of Table S9. For mechanism-guided studies, the minimal sufficient configuration may be specified provisionally before experimentation. For discovery-oriented studies, uncertain functions should remain explicit candidates and the minimal sufficient explanatory configuration should be recorded only after matched module testing and mechanistic perturbation. The record is a proposed decision aid, not a validated reporting standard. A non-significant or undetected difference is not, by itself, evidence that a module is non-essential; de-escalation requires adequate assay performance and a prespecified bound for what constitutes a decision-relevant effect.

Table S10. Revision-stage targeted scientific verification for reviewer-requested content expansion (search date: 4 September 2026).
These exact PubMed/MEDLINE queries were used to verify and expand three areas identified during peer review. Searches were run on 4 September 2026, but retained scientific records were restricted to publication or electronic publication on or before the manuscript scientific-literature cutoff of 31 July 2026. The table records the targeted verification used for revision rather than a reconstruction of all historical narrative searches.

Topic	Exact PubMed/MEDLINE verification query	Eligibility / purpose	Representative retained references
Decellularized / patient-derived ECM	(tumor OR tumour OR cancer)<br>AND ("decellularized extracellular matrix" OR "decellularised extracellular matrix" OR "patient-derived extracellular matrix" OR "patient-derived ECM" OR dECM)<br>AND (model* OR organoid* OR hydrogel* OR scaffold* OR drug response)	Verify tumour- or patient-derived dECM scaffolds/hydrogels, matrix-processing limitations, and effects on tumour state or treatment response.	[47–50]
Light-mediated bioprinting	(tumor OR tumour OR cancer)<br>AND (bioprint* OR biofabricat*)<br>AND ("digital light processing" OR DLP OR stereolithograph* OR SLA OR "light-based" OR photopolymer*)	Verify non-extrusion tumour bioprinting strategies and distinguish DLP/SLA process variables from extrusion-based fabrication.	[140–142]
Pharmaceutical exposure and drug delivery	(tumor OR tumour OR cancer)<br>AND (microphysiological OR "tumor-on-chip" OR "tumour-on-chip" OR spheroid* OR organoid* OR matrix)<br>AND ("drug delivery" OR penetration OR transport OR "delivered exposure" OR adsorption OR binding OR pharmacokinetic*)	Verify exposure, transport, penetration, binding/adsorption, and delivery-system considerations relevant to Pharmaceutics and preclinical drug evaluation.	[9,10]

Table S11. Expanded model-class reporting and quality-control checklist. This table provides the detailed variables, critical quality attributes, controls, and frequent failure modes summarized in main-text Table 5. The items are fit-for-purpose recommendations rather than universal acceptance thresholds.
Abbreviation: HLA, human leukocyte antigen.

Model class	Essential reporting variables	Critical quality attributes	Functional controls	Frequent failure mode
All patient-derived models	• Site/region; tumour content; prior therapy<br>• Collection-to-processing interval; ischaemia; transport conditions<br>• Processing; passage; cryopreservation; assay denominator; time to result	• Specimen identity; viable tumour fraction<br>• Required-compartment retention; traceability<br>• Pre-analytical robustness; stability over the decision window	• Parental/time-zero comparison<br>• Processing-delay control where feasible<br>• Compartment-specific identity checks	• Sampling/ischaemia bias; selective loss/overgrowth<br>• Culture drift<br>• Exclusion of failed/late specimens misattributed to platform biology
Multicellular spheroids	• Cell source; aggregation method; seeding density<br>• Size distribution; culture duration<br>• Exposure/washout; analysis method	• Establishment; size/compactness<br>• Baseline viability/growth; internal state<br>• Within-plate variation; assay dynamic range	• Vehicle and cytotoxic positive controls<br>• Size-matched untreated control<br>• Spatial/orthogonal viability where relevant	• Variable aggregation/gradients<br>• Segmentation error<br>• Metabolic readout misread as drug response
Tumour organoids	• Tissue source; establishment denominator<br>• Matrix/medium lot; passage; cryopreservation<br>• Intact vs reformed format; molecular identity	• Tumour-content retention; lineage/genomic stability<br>• Growth reproducibility; contamination/normal-cell control<br>• Completion within decision window	• Parental-tissue comparison<br>• Normal-organoid comparator where relevant<br>• Matrix/medium and positive-response controls	• Culture selection/clonal drift<br>• Stromal loss or normal-cell overgrowth<br>• Establishment failure/delayed reporting

Model class	Essential reporting variables	Critical quality attributes	Functional controls	Frequent failure mode
Explants / tissue slices	• Anatomical region; thickness/fragment size<br>• Processing interval; culture support<br>• Exposure route/duration/recovery; delivered-dose considerations	• Tumour content; compartment viability<br>• Tissue integrity/injury<br>• Assay-window stability; spatial response reproducibility	• Adjacent untreated tissue<br>• Time-zero histology / injury control<br>• Exposure/penetration control	• Ex vivo injury/diffusion artefact<br>• Regional sampling bias<br>• Rapid compartment loss
Matrix-engineered / bioprinted	• Material/tissue source and lot; dECM processing/residual-cell-DNA where used<br>• Composition/concentration; mechanics/crosslinking/geometry; print modality<br>• Fabrication/maturation; photoinitiator/wavelength/light dose where relevant; compound binding/recovery	• Mechanical/source reproducibility; retained ECM features<br>• Cell viability; remodelling; porosity/diffusion; shape fidelity<br>• Delivered exposure; spatial-analysis reproducibility	• Material/source controls; fabrication control<br>• Light/photocure control where relevant<br>• Remodelling perturbation; tracer/compound recovery	• Source/batch variation or incomplete/overaggressive decellularization<br>• Residual reagents/material binding<br>• Shear/phototoxic stress; imposed geometry/diffusion altering apparent potency
Tumour-on-chip / vascularized	• Device material/geometry; flow waveform/pressure/route<br>• Channel coating; cell sources; gas control<br>• Dosing route; tubing/reservoir; compound recovery	• Lumen continuity; perfusion<br>• Barrier integrity/permeability; tissue position<br>• Delivered exposure; run stability	• Tracer recovery; no-flow control<br>• Barrier perturbation controls<br>• Adsorption and pressure controls	• Adsorption/bubbles/unintended flow<br>• Hydraulic variability/leakage<br>• Non-functional endothelium
Immune-integrated	• Immune source; HLA/compatibility; antigen context<br>• Activation state; ratios; medium/cytokines<br>• Timing/route; assay duration	• Immune viability/phenotype; alloreactivity<br>• Migration/engagement/persistence<br>• Effector/suppressive function; target specificity	• Target-positive/negative<br>• Immune-only/tumour-only<br>• Autologous/allogeneic where relevant; functional blockade	• Alloreactivity; exhaustion/overactivation<br>• Cytokine/medium artefact<br>• Restricted migration/loss of function
